# Exploring Membrane Binding Targets of Disordered Human Tau Aggregates on Lipid Rafts Using Multiscale Molecular Dynamics Simulations

**DOI:** 10.3390/membranes12111098

**Published:** 2022-11-04

**Authors:** Kwan H. Cheng, Angela Graf, Amber Lewis, Thuong Pham, Aakriti Acharya

**Affiliations:** 1 Neuroscience Department, Trinity University, San Antonio, TX 78212, USA; 2 Physics Department, Trinity University, San Antonio, TX 78212, USA

**Keywords:** amyloidogenic protein, protein aggregation, neurodegenerative diseases, phase-separated lipid bilayer, neuronal membranes, MD simulations

## Abstract

The self-aggregation of tau, a microtubule-binding protein, has been linked to the onset of Alzheimer’s Disease. Recent studies indicate that the disordered tau aggregates, or oligomers, are more toxic than the ordered fibrils found in the intracellular neurofibrillary tangles of tau. At present, details of tau oligomer interactions with lipid rafts, a model of neuronal membranes, are not known. Using molecular dynamics simulations, the lipid-binding events, membrane-damage, and protein folding of tau oligomers on various lipid raft surfaces were investigated. Tau oligomers preferred to bind to the boundary domains (Lod) created by the coexisting liquid-ordered (Lo) and liquid-disordered (Ld) domains in the lipid rafts. Additionally, stronger binding of tau oligomers to the ganglioside (GM1) and phosphatidylserine (PS) domains, and subsequent protein-induced lipid chain order disruption and beta-sheet formation were detected. Our results suggest that GM1 and PS domains, located exclusively in the outer and inner leaflets, respectively, of the neuronal membranes, are specific membrane domain targets, whereas the Lod domains are non-specific targets, of tau oligomers binding to neurons. The molecular details of these specific and non-specific tau bindings to lipid rafts may provide new insights into understanding membrane-associated tauopathies leading to Alzheimer’s Disease.

## 1. Introduction

The misfolding and self-aggregation of tau, an intracellular microtubule-binding protein, has been linked to the progression of various neurodegenerative diseases, e.g., Alzheimer’s Disease [1,2]. The presence of intracellular neurofibrillary tangles (NFTs) consisting of microscopic, highly ordered and beta-sheet rich tau fibrils [3] has been established as a histopathological marker in tauopathies [1]. However, recent studies have demonstrated that the nanoscopic and highly disordered tau oligomers are more neurotoxic than the mature tau fibrils, and that the interactions of these toxic tau oligomers with neuronal membranes may play a key role in the onset of tauopathies [4]. In addition, these tau oligomers, originally created in the cytosol, may exit the neurons and translocate to other areas of the brain, including the space between the synaptically associated neurons [5]. Therefore, a molecular level understanding of tau-membrane interactions related to tau oligomers binding to the cytosolic (inner) and extracellular (outer) membrane leaflets of neurons will provide useful insights in understanding the early onset of tauopathies [1,2,6].

As a class of intrinsically disordered protein (IDP), the molecular mechanisms of misfolding and aggregation events of tau oligomers in solution and their associations with membranes are challenging to study computationally and experimentally due to the highly dynamic and heterogeneous structures of IDP [7]. Recent experimental studies have established that the lateral (along the membrane surface) and transbilayer (across the lipid bilayer) distributions of lipids in the cell membranes are not homogeneous but contain highly heterogeneous and dynamic phase-separated nanodomains, or lipid rafts [8,9,10,11] on both leaflets of the membranes.

Our model raft membranes are control raft (CO-raft), modified CO-raft containing ganglioside (GM1) clusters on one leaflet (GM-raft), and modified CO-raft containing phosphatidylserine (PS) clusters on one leaflet (PS-raft). Our model raft membranes contain phase-separated liquid-ordered (Lo) domains, liquid-disordered (Ld) domains, mixed Lo/Ld (Lod) domains in the CO-raft. In the PS-raft and GM-raft, in addition to the Lo, Ld, and Lo/Ld domains, extra transbilayer asymmetrically distributed ganglioside (GM1)-cluster domains and anionic phosphatidylserine (PS)-cluster domains are also present. The Lo, Ld, and Lod domains can be found in both inner and outer leaflets of neuronal membranes, but the GM-clusters and PS-clusters are exclusively located in the outer and inner leaflets of the neuronal membranes, respectively [8]. Therefore, our model CO-raft, PS-raft, and GM-raft are able to mimic the raft domains found on both leaflets of the neuronal membranes, the inner leaflet and the outer leaflet, respectively. How the tau oligomers interact with those coexisting domains was systematically examined in this work.

In this pilot study, we have designed and created a 130-residue long tau fragment, or K18 monomer, which represents the microtubule binding domain of tau [12], and have simulated K18 aggregates (dimer and tetramer) via self-aggregation of the K18 monomers in solution. The K18 oligomers containing 4 repeated domains (R1, R2, R3, and R4) together with the *N*-terminus are known to bind to membrane lipids, particularly the PS, and participate in beta-sheet formation upon binding [6,12]. Here, we demonstrate that K18 oligomers bind specifically to the Lod boundary domains in the CO-raft, the GM-clusters in the GM-raft, and the PS-clusters in the PS-raft mostly within 10 μs of CG simulations.

By mutating three residues near the R2 and R3 repeats of K18, significantly weaker protein-lipid binding energy of K18 oligomers to the PS raft were detected, and the results agree with the recent experimental data on K18-PS liposome binding [13]. After a CG-to-AA resolution transformation, atomistic details of protein-induced membrane disruption and membrane-templated folding of K18 oligomers were revealed. Here, significant disruptions of lipid acyl chains surrounding the membrane-bound K18 in the CO-raft and GM-raft were detected. In addition, evidence of membrane-assisted creation of transient beta-sheets of K18 on raft surfaces was found. These observations of specific membrane domain binding and damage and subsequent beta-sheet creation provide new insights into understanding membrane-related tauopathies in Alzheimer’s disease.

## 2. Materials and Methods

### 2.1. Lipid Rafts

The construction of three laterally and transversely heterogeneous, phase-separated lipid raft systems, CO-raft, GM-raft, and PS-raft were created using CG MD simulations [14]. Our raft systems contain saturated phosphatidylcholine (PC), unsaturated PC, cholesterol, monosialotetrahexosylganglioside GM1, and PS lipids, and their design was based on a fully hydrated and equilibrated 3-component coarse-grained (CG) lipid raft model [15]. Details of constructing the two rafts, CO-raft and GM-raft, are described elsewhere [16,17]. The construction of the third raft, PS-raft, was very similar to that of GM-raft in our previous studies [16,17]. Briefly, the CO-raft contains 828 saturated dipalmitoyl-PC (DPPC), 540 unsaturated dilinoleoyl-PC (DLPC), 576 cholesterol (CHOL), and 66,741 water molecules, with a lipid molar ratio of DPPC:DLPC:CHOL = 0.42:0.28:0.30 and a size of ~22 × 22 × 20 nm^3^. For either GM-raft or PS-raft, some lipids on one lipid leaflet were replaced by GM1 or 1-palmitoyl-2-oleoyl-PS (POPS) lipids. Therefore, the CO-raft contains DPPC, DLPC, and CHOL on both lipid leaflets, but the GM-raft and PS-raft contain GM1 and POPS on only one leaflet of the lipid bilayer. The molecule counts of the asymmetric GM-raft were 36 GM1, 709 DPPC, 407 DLPC, 410 CHOL, and 56,114 water molecules, with a lipid molar ratio of GM1:DPPC:DLPC:CHOL = 0.02:0.43:0.30:0.25. The counts of the asymmetric PS-raft were 162 POPS, 666 DPPC, 540 DLPC, 576 CHOL, and 65,365 water molecules, with a lipid molar ratio of POPS:DPPC:DLPC:CHOL= 0.08:0.34:0.28:0.30. The sizes of both GM- and PS-rafts were similar to that of the CO-raft.

Previous studies [15,16,17] have established that the CO-raft contains ordered DPPC-rich and CHOL-rich (Lo) domains, disordered DLPC-rich (Ld) domains, DPPC-DLPC (Lod) domains. In the presence of the asymmetrically distributed GM1 in the GM-raft, GM1-clusters were formed on one leaflet of the bilayer, with Lo, Ld, and Lod domains on both leaflets [16,17]. Similarly, the new PS-raft in this study also exhibited asymmetric transbilayer distribution of PS-clusters located on one leaflet, and Lo, Ld, and Lod were present in both leaflets as well. After 15 μs of CG MD simulations, the symmetrically distributed Lo, Ld, and Lod domains as well as the asymmetrically distributed GM-cluster and PS-cluster domains were still present, and these lipid rafts were used as the initial membrane structures for the protein binding studies.

### 2.2. Tau-K18 Oligomers

**Figure 1:**
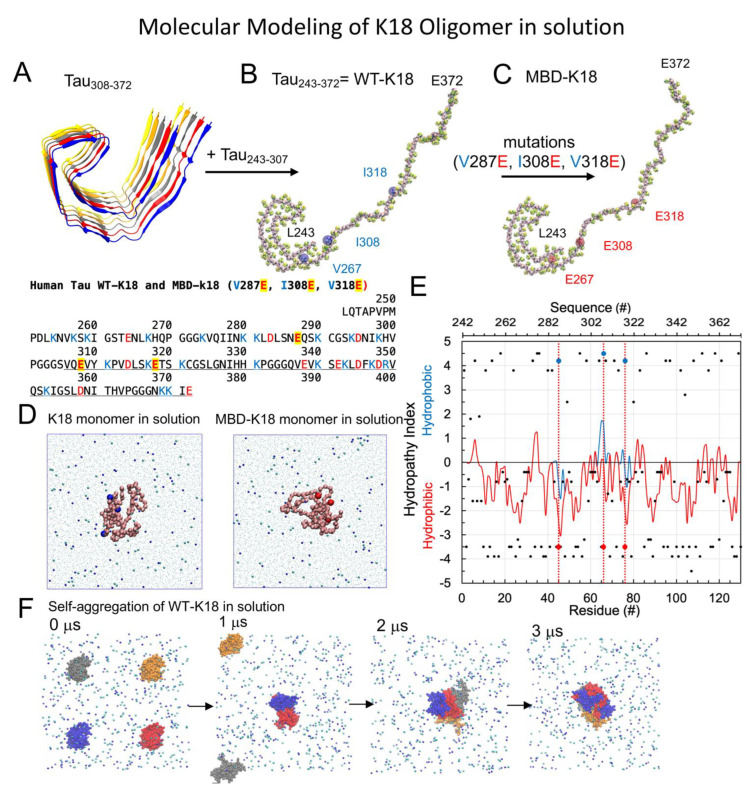
Tau-K18 oligomers in solution. The chain A (blue ribbon) from the CryoEM all-atom (AA) tau pentamer structure (**A**) was used as a template (underlined in the primary sequence) to build the AA wild type K18 or WT-K18 (**B**) and mutated K18, MBD-K18 (**C**) with the three mutation sites identified. The coarse-grained (CG) structures, yellow beads for side chains and pink beads for backbones, of both monomers are overlaid to the AA structures (in licorice). The backbone structures of monomeric K18 peptide after 5 μs of CG simulations in 0.1 M NaCl, with water shown as small dot, Na^+^ in dark blue sphere, Cl^−^ in light blue sphere, and the residues before (blue) and after (red) mutations are shown (**D**). The hydropathy plot of K18 monomers, i.e., hydropathy index vs. residue or sequence number with a 5-point moving average fit, for WT-K18 (blue) and MBD-K18 (red), is given (**E**). The self-aggregation process of four WT-K18 monomers, with each chain in a different color (blue for chain A, red for chain B, gray for chain C, and orange for chain D) within 3 μs of CG simulation in 0.1 M NaCl is demonstrated (**F**).

Wild-type and mutated Tau-K18 monomers were constructed using MD simulation procedures. Tau-K18 is a truncated Human Tau peptide containing four microtubule binding repeats [6]. The creation of an all-atom (AA) 130-residue long Tau-K18 monomer was based on a cryoEM-derived, fibrillar tau pentamer structure [3] as shown in Figure 1A. Here, a 73-residue long peptide Tau_308-372_ was first extracted from the chain A of the pentamer structure. Thereafter, a 57-residue long random coil Tau_243-307_ was attached to the *N*-terminus of Tau_308-372_ using a homology modeling algorithm [18] to create the final 130 residue-long Tau_243-372_, or the wild-type Tau-K18 (WT-K18) monomer. A membrane-binding deficient (MBD) mutant [13] of Tau-K18, or MBD-K18, was created by mutating three highly hydrophobic residues at V287, I308, and V318 to three negative residues at E287, E308, and E318. The mutations at V287E, I308E, and V318E are highlighted in both the primary and AA structures of WT-K18 and MBD-K18 (Figure 1A–C).

A hydropathy index vs. residue number plot [19,20] was also used to characterize the hydrophobicity profile of the amino acids of the two K18 monomers. The hydropathy plot revealed significant drops in the hydropathy indices from ~0 to −3, 2 to −2, and 0 to −3 at the three mutation sites, respectively, as shown in Figure 1E. As shown in Figure 1A, the amino acid charge counts of WT-K18 are +21e and −11e, with a net charge of +10e, while the counts of MBD-K18 are +21e and −14e, with a net charge of +7e. Therefore, WT-K18 also carries more positive charge than MBD-K18. Supplementary Materials Figure SA1 further demonstrates the R1, R2, R3 and R4 repeats of WT-K18 and MBD-K18. A close examination of the primary sequences reveals that the first mutation V287E is at the middle of R2 and the other two mutations, I308E and V281E, are near the beginning and the middle of R3 repeat.

CG monomers were created from the corresponding AA structures from above using the AA-to-CG resolution transformation program *martinize.py* [21] based on the MARTINI-2.20 CG force fields [22]. Since no secondary structures were present in the AA monomer, no external dynamic network or constraints were imposed on the atoms of the monomers. The CG monomers were solvated in 0.1 M NaCl, and underwent energy minimization and position-restraints to reduce the local high energy structure created during the solvation steps. Finally, the monomer in solution underwent a 5 μs-long CG MD simulation in the NPT ensemble using the Martini CG force fields [22] and ran on the GROMACS-5.1.5 MD simulation program [23]. Detailed CG-simulation procedures for simulating disordered monomers in solution can be found [16,17]. The final 5 μs monomer structures of WT-K18 and MBD-K18 were used to create dimer and tetramer.

To generate the self-aggregated CG Tau-K18 dimer and tetramer, the above monomers were self-replicated along different *x*-, *y*- and *z*-directions using the molecule replication tool *genconf* of GROMACS [23], followed by the same MD simulation procedure as above to model the self-aggregation process. The procedure was identical to the self-aggregation process published recently [17]. Briefly, a dimer was created by replicating the simulation box containing a single monomer, or monomer-self-replication, along the *x*-direction, to generate two separated monomers lined up along the *x*-direction in a larger simulation box (see Figure SC1). Similarly, a tetramer was generated by monomer-self-replication along three directions, *x*, *y* and diagonal *x*-*y* directions, as shown in Figure 1F.

To evaluate the protein conformations of oligomers, 3D protein residue-contact maps describing the color-coded residue-residue minimum distances, among all protein residues, along the *x*- and *y*-axes, were calculated using the *g_mdmat* tool of GROMACS [23] and a statistical and molecular interaction analysis tool, CONAN [24]. Time-averaged protein residue-contact maps with standard deviation were generated. Details of residue-contact map generation were described in our previous studies [17,25].

### 2.3. CG Simulations of Oligomer-Lipid Raft Complexes

Incorporation of the CG oligomer (monomer, dimer, or tetramer) into the equilibrated CO-raft, GM-raft, and PS-raft was accomplished by placing each oligomer above the center of the upper lipid leaflet of each lipid raft using the *genbox* tool of GROMACS [23]. Some solvent atoms were removed to accommodate the newly added protein. In this study, three independent simulation replicates corresponding to three different initial positions of each oligomer placed above the surface of the lipid raft were created. The first replicate was placed at 5 nm above the center of the lipid leaflet, and the other two replicates were subsequently created with the protein position shifted ±2 nm along the *x*-direction relative to the position of the first replicate.

The use of three independent simulation replications for each oligomer-raft complex is important to improve phase sampling of protein-membrane binding events. Upon generating the initial structures of the three replicates, the same CG MD simulation procedures for oligomer simulations were performed for up to 15 μs or longer.

In this study, a total of 54 simulation replicates involving oligomers of 3 aggregation sizes, 3 lipid rafts, and 3 simulation replicates for each oligomer-lipid raft complex with an accumulated CG simulation time over 800 μs were generated. Since the CG simulation time based on the Martini force field is roughly four times faster than the real time based on the CG diffusion rate of water [15,22], this study may represent more than 3 milliseconds of biological processes that are relevant to the early aggregation of proteins on membrane surfaces.

### 2.4. AA Simulations of Oligomer-Lipid Raft Complexes

After the CG simulations of oligomer to lipid raft binding, each replicate of the CG tetramer raft system was converted to the AA structure using a CG-to-AA resolution transformation program, *backward.py* [26]. The newly transformed AA structure was equilibrated with similar energy minimization and position restraining procedures as in the simulations of the CG oligomer-raft complexes. However, instead of the Martini CG force fields, the atomistic AMBER99SB [27] for proteins and SLIPID [28,29,30,31] for lipids force fields were used in all AA simulations.

### 2.5. Characterizations of Phase-Separated Lipid Domains and Annular Lipids

A data-filtering tool, *g_select*, from GROMACS [23] was employed to classify lipids into three phase-separated domains: Lo, Ld, and Lod. The Lo domain represents the DPPC-rich domain. In this Lo domain, at least one atom of each DPPC lipid is within 0.5 nm of the atoms of another DPPC lipid. Similarly, the Ld domain is the DLPC-rich domain in which one atom of each DLPC is within 0.5 nm of the atoms of another DLPC lipid. The Lod domain consists of DPPC molecules that are not included in the Lo domain and DLPC molecules that are not included in the Ld domain. Finally, Lo-CHOL, Ld-CHOL, and Lod-CHOL represent distinct groups of CHOL, for which at least one CHOL atom is within 0.5 nm of the PC lipid atoms in Lo, Ld, and Lod domains, respectively. The same *g_select* tool as above was also used to characterize annular lipid shells from each oligomer/raft system. Here, if an atom of any lipid (DPPC, DLPC, CHOL, or GM1) is within a certain threshold, e.g., 0.5 or 1.2 nm from an atom of an oligomer, that lipid is assigned to the 0.5 nm or 1.2 nm annular lipid shell, accordingly.

The time- and replicate-averaged number of lipids in each lipid domain or annular lipid shell over the last 5 μs for CG and 50 ns for AA simulation were performed to assess the domain or annular shell compositions of our raft systems in the absence and presence of membrane-bound K18 oligomers. Additionally, the lipid orientational order parameter, a measurement of the tilt of three sequentially connected carbon atoms along the lipid acyl chains of DPPC, DLPC, POPS and GM1 with respect to the normal of the bilayer, was calculated as a function of the carbon number of lipid acyl chain. Detailed steps of characterizations of composition and lipid order parameters of the lipid domains and annular lipids are described elsewhere [16,17,25].

### 2.6. Membrane Binding Kinetics, Domains, and Energetics of Oligomers

Examination and visualization of the binding kinetics related to the time events of protein-membrane binding and the lipid types surrounding the membrane-bound oligomers for each replicate were performed using a molecular visualization program, VMD [32].

Statistical analysis of the lipid-binding kinetics and locations of the protein binding were obtained using the minimum-distance analysis tool, *g_mindist*, from GROMACS [23]. Briefly, a protein-lipid or protein-water minimum distance (*mindist*), defined as the minimum distance between any protein atom and the atom of its binding lipid or water neighbors, was determined as a function of simulation time. In addition, the number of contacts of the *mindist* within an interaction threshold (2 nm) vs. simulation time was also determined. Finally, the time-averaged *mindist* vs. protein residue number over the last 5 μs of the 15 μs long CG simulation or the last 50 ns of the 200 ns long AA simulation was calculated. These three parameters, *mindist* vs. time (upper panel), number of contacts vs. time (mid panel), and *mindist* vs. residue number (bottom panel) were expressed as a 3-panel plot of each replicate. The first two panels were used to determine the kinetics of protein binding in terms of the time event of protein attachment, or lipid binding time, to each lipid type. The last plot provides important information about the *mindist* of the nearest-neighbor lipids or water surrounding the protein upon forming a membrane-bound state.

These *mindist* analysis plots and the VMD analyses provide the basis for determining the lipid kinetics and binding regions of K18 oligomers and for establishing the membrane domain target of the membrane-bound oligomer in the model raft.

Similarly, the time-averaged and replicate-averaged nonbonded interaction energies between the oligomer and each lipid group, or protein-lipid binding energy were collected using the tool, *g_energy*, from GROMACS [23] for each oligomer/raft complex. Both nonbonded Van Der Waals or Lennard-Jones and electrostatic or Coulomb energies were separately calculated from CG and AA simulations of our protein/raft systems. Details of the use of the above analysis tools for studying protein/raft binding kinetics, behaviors and energetics are described elsewhere [16,17].

### 2.7. Secondary Structure Determination

The secondary structure of each amino acid of the protein at every time-step was calculated using a tool, *do_dssp*, from GROMACS [23] based on the Define Secondary Structure of Proteins (DSSP) algorithm [33]. DSSP is the standard method of identifying secondary structures, and it is based on electrostatic energies among hydrogen donors and acceptors of the protein. Eight types of secondary structure are assigned, including hydrogen-bond based structures (helical, beta sheet, and turn) as well as non-hydrogen-bond based structures (bend and coil). These structures are color coded and represented in a 3D map with the *x*-axis indicating the simulation time and the *y*-axis the residue location. Note that VMD was used to visualize the 3D image of the protein secondary structure. However, the protein structure calculation from VMD was based on the STRIDE algorithm [34] which is slightly different from DSSP. The assessment of membrane-induced protein folding, or disordered-to-order transition, of K18 was based on the DSSP only.

## 3. Results

### 3.1. CG-Tau Oligomers in Solution

As described in Materials and Methods, CG WT-K18 and CG MBD-K18 oligomers were created by a self-aggregation process by bringing multiple (two or four) monomers near each other in a simulation box to create the initial structure. Oligomers were then created after performing CG MD simulations of the monomers in 0.1 M NaCl, at 310 K and 1 atmospheric pressure. Figure 1F demonstrates the highly dynamic nature of the aggregation process during the first 3 μs of the self-aggregation process starting from four monomers. Similar observation was found for the formation of the dimer as demonstrated in Supplementary Materials Figure SC1. When compared with tetramer, a dimer was readily formed in less than 1 μs (Figure SC2A), but it took around 2 μs to establish an aggregated tetramer. Similar self-aggregation rates were found for the MBD-K18 in solution. After the protein self-aggregation process, the binding kinetics, domain preference and energetics of these pre-formed oligomers on different lipid rafts were systematically investigated.

Using a statistical and molecular interaction analysis tool, CONAN (see Materials and Methods), Supplementary Materials Figure SB1 shows the time-average and standard deviation protein-residue contact maps of monomeric and tetrameric WT-K18. It is clear that both monomer and tetramer exhibit large residue-contact regions as shown in the average contact maps and large fluctuation of residue-contacts as reflected in the standard deviation of contacts among the residues in the protein.

### 3.2. CG-Tau Oligomers Binding to Lipid Rafts

Three different lipid rafts, CO-raft (Figure SC2A), GM-raft (Figure SC2B) and PS-raft (Figure SC2C), in 0.1 M NaCl were used as model membranes. In the absence of an externally added protein, structurally heterogeneous, and highly dynamic lipid domains, DPPC-rich Lo (light green), DLPC-rich Ld (light orange), and mixed DPPC-DLPC Lod (dark green or dark orange) were observed. Figure SC2A demonstrates the domain structures of these Lo, Ld, Lod on both leaflets of the bilayer in both lateral and transverse views of the CO-raft. Figure SC2B also shows the GM1-clusters on one leaflet of the bilayer. Similarly, the PS-clusters which were found on Lo and Lod domains but not in the Ld domains on one side of the leaflet are demonstrated in Figure SC2C. The cholesterol was mainly found in the Lo domains in both lateral and transverse views for all rafts.

Using the *g-select* tool of GROMACS as mentioned in Methods, the lipid compositions of lipids in the Lo, Ld and Lod domains were determined, and the results are summarized in Figure SC5. For the cholesterol, about ~60% of CHOL were distributed to the Lo domains vs. ~10% and 30% to the Ld and Lod domains, respectively, for the CO-raft and GM-raft. For the PS-raft, the distributions of CHOL were ~50%, 15% and 35% in the Lo, Ld and Lod, respectively. Therefore, the presence of the GM-clusters had no effect on the CHOL distributions in Lo, Ld and Lod domains. However, the presence of PS-clusters slightly reduced the CHOL partitioning to the Lo domains and concomitantly increased its partitioning to the Ld and Lod domains. The DPPC % in the Lo domain and DLPC % in the Ld domain reflect the relative sizes of the Lo and Ld domains in each raft. Here, Lo-DPPC% were ~20% in all rafts, and Ld-DLPC ~10%. Furthermore, the total Lod % which is the sum of Ld-DPPC% and Lo-DLPC% accounted for ~70%. Therefore, our raft systems provided the dynamically stable Lo, Ld and Lod domains, as well as the asymmetrically distributed GM-clusters and PS-clusters, to examine the lipid domain-binding preference of tau oligomers on raft surfaces. Protein-residue contact maps (Figure SB2) of monomeric and tetrameric WT-K18 bound to CO-raft revealed smaller residue-contact regions and fluctuation of residue-contacts were evident when compared to the protein in solution.

**Figure 2:**
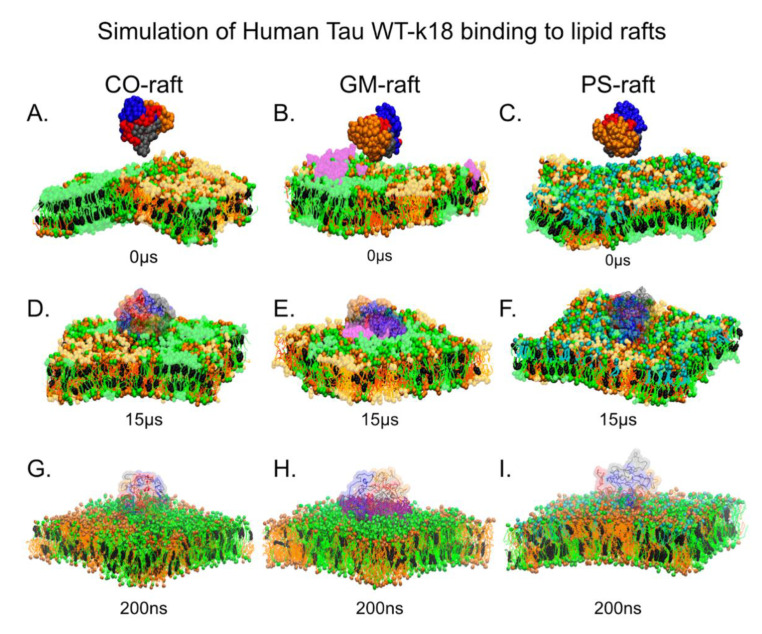
Tau WT-K18 tetramers in lipid rafts. (Panels (**A**–**C**)). The beginning structures (0 μs) of our simulation systems consisting of coarse-grained (CG) WT-K18 tetramers placed at ~5 nm above the center of each CG lipid raft. (Panels (**D**–**F**)). The final 15 μs structures of the CG systems after CG MD simulations. Both CG and all-atom (AA) structures (after the CG-to-AA transformation) of tetramers are overlaid. (Panels (**G**–**I**)). The final 200 ns AA structures of the systems after AA MD simulations. DPPC lipids are in green with DPPC-enriched regions (Lo) in a lighter color. DLPC lipids are in orange with DLPC-enriched regions (Ld) in a lighter color. POPS-clusters are in cyan, and GM1-clusters are in light purple. The CG tetramers are shown in color beads with chain A in blue, chain B in red, chain C in gray and chain D in orange. The AA tetramers are presented in color ribbons. All simulations were performed in 0.1 M NaCl at 310 K and 1 atmospheric pressure.

To investigate the binding behaviors of tau oligomers on raft surfaces, the pre-formed, self-aggregated oligomers were placed at different positions (see Materials and Methods) in solution to create the starting or 0 μs structures of the CG oligomer and raft simulation replicates. Figure 2 demonstrates the binding behavior of tetramers starting from the solution states and above the surfaces of the CO-raft (Figure 2A), GM-raft (Figure 2B) and PS-raft (Figure 2C) to the final equilibrated, membrane-attached or -bound states after 15 μs as shown in Figure 2D–F, accordingly. Figures SC2 and SC3 demonstrate the similar tau dimer binding behaviors to the CO-raft, GM-raft and PS-raft from the solution states to the membrane-bound states.

The tau oligomer, both WT-K18 and MBD-K18, bound to the Lod domain in the CO-raft, and the asymmetrically distributed GM-cluster or PS-cluster on one leaflet of the bilayer. For the PS-raft binding, all three independent replicates of tetrameric WT-K18 and MBD-K18 bound exclusively to the PS-clusters within 6 μs. However, one replicate of monomeric WT-K18, one replicate of dimeric WT-K18 and one replicate of monomeric MBD-K18 bound to the leaflet in the absence of PS. In other words, the protein bound to the opposite leaflet, where there were no PS lipids present. In those systems, an extra replicate, or fourth replicate, with a slightly different initial position from the raft surface, was created, and successful PS-cluster binding of the fourth replicate was achieved. This observation suggests that the binding affinity to PS-clusters and Lod domains may be similar for the small tau oligomers. In contrast, all oligomers bound to GM-clusters and no non-specific binding to the “opposite leaflet” were observed in both WT-K18 and MBD-K18. The results further indicate that binding affinity to GM-clusters may be stronger than the binding affinity to Lod and PS-cluster domains, for tau oligomers of all sizes.

No significant disruptions of the domain organization were detected in all our oligomer binding studies. Figure SC5 shows the time- and replicate-averaged domain compositions over the last 5 μs and across all simulation replicates in the presence of membrane-bound monomeric (*n* = 1), dimeric (*n* = 2) and tetrameric (*n* = 4) of CG WT-K18 and CG MBD-K18. No significant changes of the domain compositions were evident before and after the protein attachment for both CG WT-K18 and CG MBD-K18. These results indicate that the lateral organization of the raft domains was unaffected by membrane-bound oligomers.

At the end of the CG simulations, a CG-to-AA resolution transformation step was carried out to convert the final CG structures to the starting AA structures, as demonstrated by overlaying the CG and AA structures in Figure 2D–F. Each converted AA structure then underwent 200 ns of AA simulation, and the final 200 ns AA structures of the oligomer/raft complexes are shown in Figure 2G–I. All tau oligomers remained firmly attached to the raft surfaces during the AA simulations.

### 3.3. Lipid Binding Analysis of K18 Oligomers

**Figure 3:**
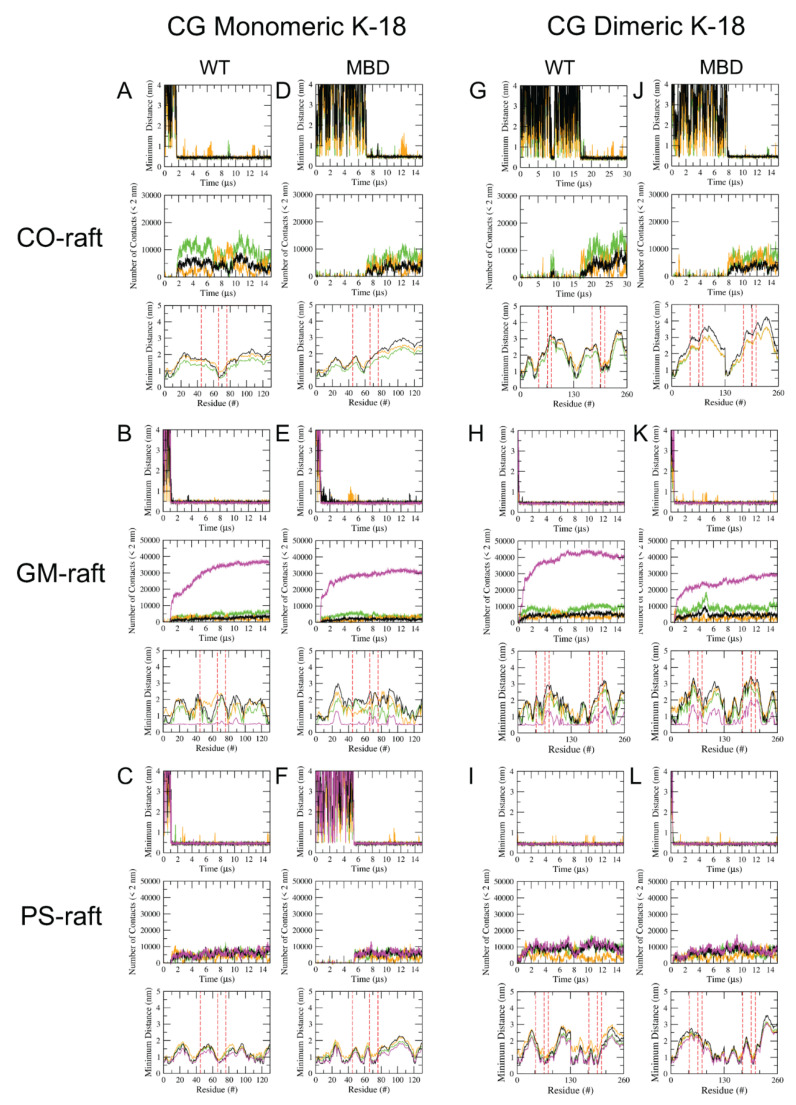
Minimum distance analysis of CG Tau oligomers binding to lipid rafts. Three-panel plots of protein-lipid minimum distance (*mindist*) of CG K-18 monomer and dimer binding to CO-raft (**A**,**D**,**G**,**J**), GM-raft (**B**,**E**,**H**,**K**) and PS-raft (**C**,**F**,**I**,**L**). For each of lipid rafts, the upper panel shows the *mindist* vs. time, the middle panel shows the number of contacts for protein and lipid atoms within 2 nm vs. time, and the lower panel shows the time-averaged *mindist* vs. residue number over the last 5 μs. All *mindist* data points are color coded according to the lipid types, DPPC in green, DLPC in orange, CHOL in black and GM1 or POPS in magenta. The red dashes are mutation markers (see text for details).

A minimum distance (*mindist*) analysis (see Materials and Methods) based on a 3-panel *mindist* plot format was performed to investigate the binding kinetics and residue-specific binding domain of K18 oligomers on all three rafts. Figure 3 shows the *mindist* plots for the representative replicates of CG monomers and dimers of WT-K18 and MBD-K18 on all lipid rafts, and Figure SD1 in Supplementary Materials shows the plots for the representative replicates of CG tetramers and AA tetramers of WT-K18 and WT-K18 for all lipid rafts.

To study the binding kinetics, the *mindist* vs. time (upper panel of the 3-panel plot) was used to quantify the lipid-binding time of CG K18 oligomers to raft surfaces. Here, the lipid binding time is defined as the time at which the protein undergoes a transition from the solution state with a large *mindist* value to a stable, membrane-bound state with a small and stabilized *mindist* value. For example, abrupt declines or transitions in *mindist* were detected at 1.72 μs and 7.63 μs for the WT-K18 monomer and tetramer in the CO-raft, respectively, as demonstrated in Figure 3A,D.

Table 1 summarizes the lipid-binding time for all 54 independently simulated CG replicates. In general, for the CO-raft, the binding times varied over a wide range from 0.1 to 20 μs. All K18 monomers in the CO-raft established stable membrane binding within 10 μs; however, the K18 dimers, particularly the WT-K18 dimers, experienced longer binding times, ranging from 14 to 20 μs. On the other hand, the binding times of K18 to PS-raft were within 6 μs, and those to GM-raft were even shorter, i.e., within 3 μs. These results suggest stronger or more efficient binding of tau oligomers to the anionic GM- and PS-rafts than to the CO-rafts.

**Figure 4:**
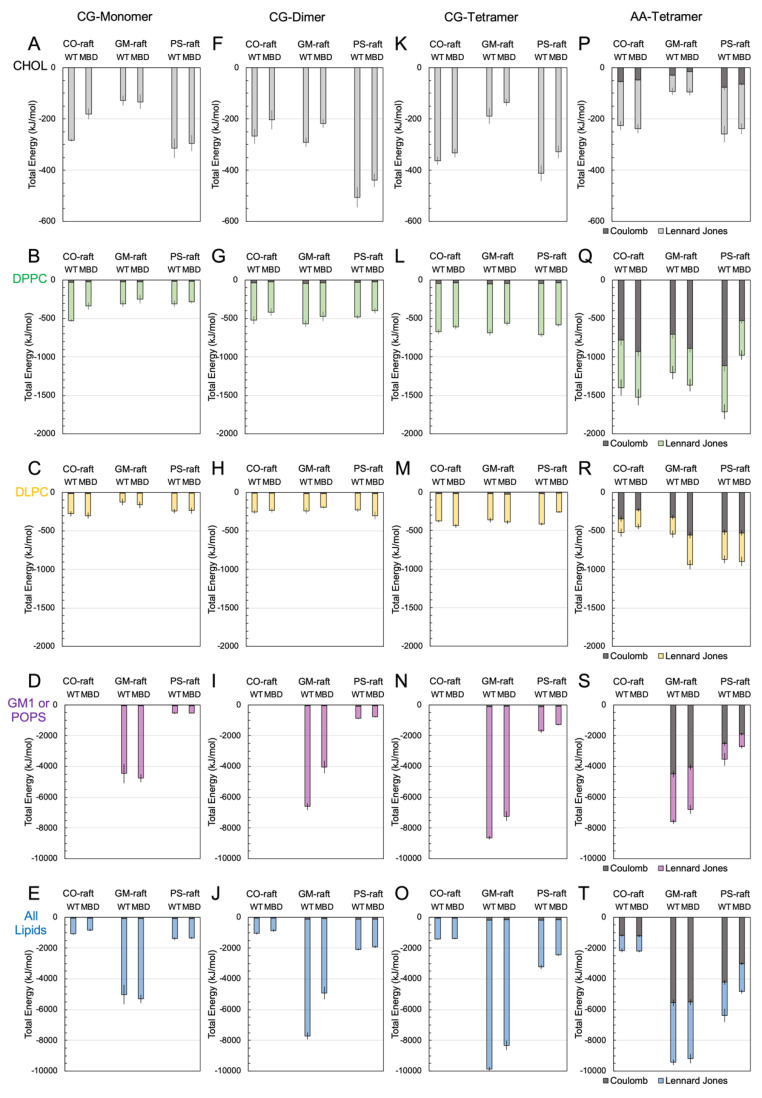
Protein-lipid binding energy of Tau-K18 oligomers. Protein-lipid binding energy of CG-monomeric (**A**–**E**), CG-dimer (**F**–**J**), CG-tetrameric (**K**–**O**) and AA-tetramer (**P**–**T**) of WT-K18 and MBD-K18 on CO-raft, GM-raft and PS-raft surfaces. Each data point represents the time- and replicate-averaged binding energy over the last 5 μs for CG-oligomers and 50 ns for AA-oligomers and across all independent simulation replicates with the error bar indicating the standard error of the mean. Both Coulomb and Lennard Jones interactions are shown. All data point sets are color coded based on the lipid types with CHOL in black, DPPC in green, DLPC in orange and GM1 or POPS in magenta. The sum of binding energies from all lipids in each lipid raft is also given (**E**–**T**).

The number of contacts vs. time plots (mid-panel of the 3-panel plot) was used to investigate the lipid environment surrounding the membrane-bound oligomers. As expected, the number of contacts between protein and lipid atoms increased with the size of the oligomer, i.e., from monomer to tetramer, for each lipid raft, as demonstrated in Figure 3 and Figure 4. Additionally, the number of contacts between the protein and GM1 lipids in the GM-raft (Figure 3B,E) was about three times more than those between the protein and other non-GM1-lipids in the same GM-raft, as well as in other rafts, i.e., CO-raft (Figure 3A,D) and PS-raft (Figure 3C,F). The observation suggests that GM1-lipids provide a more stable binding environment and a larger number of protein binding sites to K18 than other non-GM1 lipids in all three lipid rafts.

Other than the number of contacts data, Table 1 shows the time- and replicate-averaged lipid compositions and the total number of lipids in the 0.5 nm annular lipid (AL) shell that provide a quantitative evaluation of the lipid environment of the membrane bound-protein. Overall, the total number of lipids within the 0.5 nm AL shell increased steadily as the size of the oligomer increased. Additionally, the oligomers of all sizes bound to the Lod domain in CO-raft, GM1-clusters in the GM-raft, and PS-clusters in the PS-raft. For the CG-system, the number of lipids in the AL shells for the WT-K18 oligomers was greater than those for the MBD-K18 oligomers, suggesting a stronger affinity of WT-K18 to GM1 when compared to MBD-K18. Yet, no significance difference was found in the AA-system.

To investigate the residue-resolved lipid-binding conformation of oligomers, the time-averaged *mindist* over the last 5 μs of simulation vs. protein residue number, or *mindist* spectrum, of each simulation system was examined (SI-D). Due to the presence of multiple chains, accumulated residue numbers, i.e., 1–130, 131–260, 260–390 and 391–520, are used to label the residue locations A–D, accordingly. The locations of mutations are marked with dashed red lines in the plots, specifically at 45*, 66* and 76* for chain A, 175*, 196* and 206* for chain B, 305*, 362* and 336* for chain C, and 435*, 492* and 466* for chain D. Note that any dips or minima in the *mindist* vs. residue number plots reflect strong affinity for the protein at those residues where the dips are found. For reference, only dips with *mindist* less than 1.0 nm were considered.

A difference spectrum (WT-K18–MBD-K18) represents an intuitive way to look at how WT-K18 and MBD-K18 differ in preference of residue-lipid contacts. It is more helpful to consider replicate-averaged *mindist* than chain-averaged *mindist*, because the conformational geometry of larger oligomers (i.e., tetramers) prevents all chains from equally accessing the membrane. Even without chain-averaged results, the *mindist* spectra of higher order oligomers are too convoluted to track meaningful protein-residue binding preferences which are otherwise obvious in monomers and dimers.

Our results reveal that the point mutations significantly decrease the K18’s binding affinity and stability in the CO-raft across all sizes of oligomers. For example, the difference spectrum plot of the K18 monomer in the CO-raft as shown in Supplementary Materials Figure SD2 demonstrates a clear dip between the second mutation at residue 66* and the third mutation at residue 76*. The *mindist* plots of K-18 monomers (Figure 3A,D) reveal that the WT-K18 binds closely to the membrane (about 0.5 nm) at the second point mutation, residue 66*, whereas an opposite effect is evident with the MBD-K18. The only region where the MBD-K18 oligomers exhibit clean and consistent binding is at the *N*-terminus of each chain, a prominent binding site across all oligomer types and sizes.

For the GM-raft, most residues in both WT-K18 and MBD-K18 interacted strongly with the GM1 lipids. Figure 3 and Supplementary Materials Figure SD3 show that the minimum distance between the WT-K18 and the GM lipid maintains a relatively flat baseline of about 0.5 nm, with small peaks around residues 73 and 90. Conversely, the MBD-K18 monomer exhibits a less stable baseline, also at about 0.5 nm, but with several small peaks from residues 20–30, 66*, 73, and 90–100. Overall, the point mutations appear to have little effect on the protein binding pattern in the presence of GM1 lipids; however, the distances between protein and membrane components do indicate that the WT-K18 binds more closely to GM1-clusters than does the MBD-K18.

For the PS-raft, both the replicate-averaged WT-K18 monomer and dimer appear to bind more closely with the PS membrane than the MBD-K18, as shown in Figure 3. The *mindist* difference plot of the monomer shows a clear dip at residue 66*, as does the chain B of the dimer at residue 196* (Supplementary Materials Figure SD4). Notably, the minimum distances between the protein and water molecules also exhibit significant peaks at these points, up to about +0.15 nm. These results suggest that (a) the non-mutated, hydrophobic residues 66* and 196* represent the major binding sites of WT-K18, and (b) the mutated, negatively charged residues of MBD-K18 bind poorly to the anionic PS-lipid domains relative to the other rafts.

Due to the limited phase sampling behaviors of AA simulations, extensive differences in the protein-lipid binding patterns in AA vs. CG are not expected. However, in this work we observed subtle differences in protein-binding patterns in asymmetric rafts. In general, we observed a stronger protein-lipid interactions in AA simulations vs. CG simulations at various regions, e.g., residue 130 of MBD-K18 on GM-raft (Supplementary Materials Figure SD6), residues 76* and 435* of WT-K18 on PS-raft, and residues 42, 492* of MBD-K18 on PS-raft (Supplementary Materials Figure SD7). Certainly, much longer AA simulation times are needed to explore the detailed protein-lipid interactions of K18 on raft surfaces.

### 3.4. Protein-Lipid Interactions of Tau Oligomers

Protein-lipid binding energies were calculated to evaluate the binding affinity of tau oligomers with each lipid type, and the results are shown in Figure 4 for oligomers of different sizes at CG and AA resolutions. All binding energies represent both the time-average and replicate-average over the last 5 μs for CG or 50 ns for AA, and across all three independent replicates. Here, nonbonded Lennard-Jones and Coulomb (electrostatic) binding energies and their sum (total binding energy) were determined. For the CG-oligomers of all sizes, the Coulomb energy was much less than 10% of the total protein-lipid binding energy. In contrast, for the AA-oligomers, the Coulomb energy was made up of >50% and ~20–30% of the total protein-lipid binding energy for diacyl lipids (Figure 4Q–S) and cholesterol (Figure 4P), respectively. This difference is attributed to the difference in the fine and extensive partial charge distribution of the atomistic structure of the protein and lipid molecules in the AA force fields vs. the CG force fields.

The protein-cholesterol binding energy of WT-K18 was generally stronger than that of MBD-K18 for CG-oligomers of all sizes in all three lipid rafts (Figure 4A,F,K), with the largest energy being present in the PS-raft. No significant difference was found between WT-K18 and MBD-K18 for AA-tetramers (Figure 4P).

The protein-DPPC binding energy of WT-K18 was also stronger than that of MBD-K18 for the CG-oligomers of all sizes in all rafts (Figure 4B,G,L). For the AA-tetramer, the protein-DPPC binding energy of MBD-K18 was slightly higher than that of WT-K18 in CO-raft and GM-raft, but the binding energy of WT-K18 was almost twice as large as that of MBD-K18 in PS-raft (Figure 4Q), indicating a significantly stronger binding affinity of WT-K18 to DPPC when compared with MBD-K18 at both CG and AA resolutions. In addition, in the AA resolution, the Coulomb energy between protein and DPPC for the WT-K18 was twice as large as that for the MBD-K18, indicating a strong electrostatic interaction between DPPC and WT-K18 protein in the PS-raft.

For DLPC, the protein-lipid binding energy was overall smaller than that for other diacyl lipids for both CG- and AA-oligomers. Interestingly, the protein-DLPC binding energy of MBD-K18 was twice as large as that of WT-K18 for the AA-oligomers in GM-raft as shown in Figure 4R.

For both the GM1 and POPS lipids, WT-K18 consistently exhibited greater protein-lipid binding energies than MBD-K18 across all dimers and tetramers at both CG and AA resolutions, indicating that the WT-K18 oligomers were more strongly attracted to the anionic GM1 and PS domains than other lipid types. In addition, the binding energy between the protein and GM1 was almost 6–10 times stronger than the that between the protein and non-GM1 lipids, e.g., the protein-GM1 binding energy of ~−8000 kJ/mol vs. the protein-DPPC binding energy of ~−1200 kJ/mol for the AA-tetramer on the same GM-raft surface. Similarly, the protein-POPS binding energy (~−3000 kJ/mol) was about 2 times stronger than that of the protein-DPPC (~−1200 kJ/mol) for the AA-tetramer on the same PS-raft surface.

Since the protein-lipid binding energy depends strongly on the number of lipids surrounding the protein, we have also calculated the normalized protein-lipid binding energy by dividing the total binding energies by the number of annular lipids with a cutoff-distance of 1.2 nm (Supplementary Materials Figure SE1). The choice of 1.2 nm reflects the cut-off energy calculation threshold, as described in previous studies [16,17]. The resulting normalized protein-lipid binding energy plots are shown in Supplementary Materials Figure SE2. The normalized protein-lipid binding energies for CHOL, DPPC and DLPC were within ~10 to ~40 kJ/mol, and no significant differences were found between protein-lipid binding energies of WT-K18 and MBD-K18 at both CG and AA resolutions, with the exception that the protein-DPPC binding of WT-K18 tetramer was much greater than that of the MBD-K18 tetramer in the PS-raft at the AA resolution (Supplementary Materials Figure SE2N), similar to the trend in total binding energy (Figure 4Q). Our normalized protein-lipid binding results further reveal the ranking order of the lipid binding affinity of tau-oligomers according to the lipid type. The order is GM1 >> PS > DPPC > DLPC > CHOL.

### 3.5. Structure of Annular Lipids Surrounding Tau Oligomers

**Figure 5:**
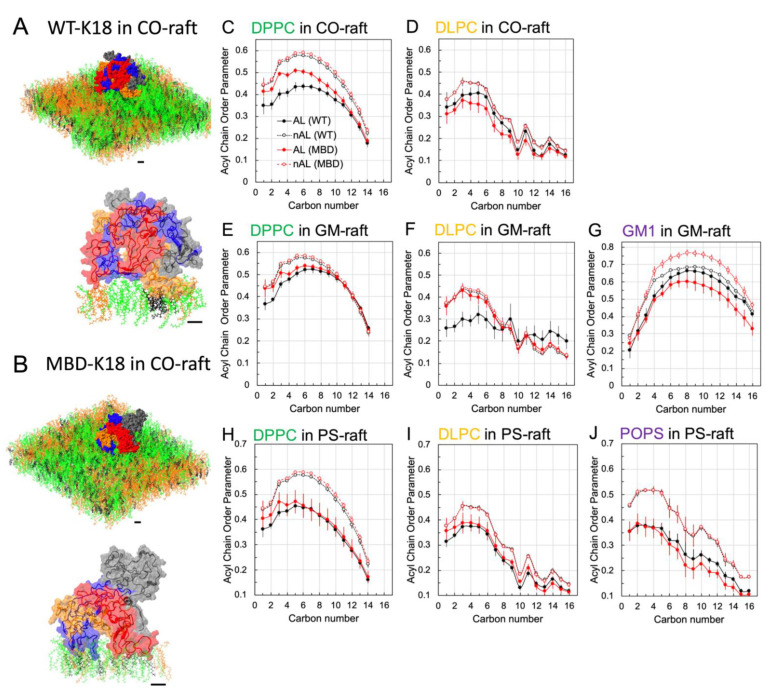
Orientational order parameter of 0.5 nm annular lipids. The membrane-bound WT-K18 tetramer and MBD-K18 tetramer on the surface of CO-raft as well as the 0.5 nm annular lipids (lower panels) surrounding the membrane-bound tetramer in CO-raft are shown. The selections of the AL shells were based on the distances between the atoms of AA-WT-K18 or AA-MBD-K18 tetramer and lipids that are within 0.5 nm (see Materials and Methods). The protein surfaces are color coded with chain A in blue, chain B in red, chain C in gray, and chain D in orange. The secondary structures are shown in the lower panels of (**A**,**B**). Lipid orientational order parameter vs. carbon number of the acyl chain of DPPC, DLPC, GM1 and POPS in CO-raft (**C**,**D**), GM-raft (**E**–**G**) and PS-raft (**H**–**J**) in the annular lipid (AL) and non-annular lipid (nAL) shells are shown. The nAL shells contain lipids that are excluded from the AL shells. Each data point represents time- and replicate-averaged over the last 50 ns and across all independent simulation replicates. For diacyl chain lipids, orientational parameters from both sn-1 and sn-2 at each carbon number were combined in the calculations. The scale bar indicates 10 angstroms.

The time- and replicate-averaged lipid orientational order parameters vs. carbon number of 0.5 nm AL lipids surrounding the AA WT-K18 and AA MBD-K18 tetramers over the last 50 ns and across three independent simulation replicates were calculated (Figure 5). Here, the 0.5 nm-AL lipids are selected according to the minimum distances of the atoms of the proteins and lipids that were within 0.5 nm (see Materials and Methods). Figure 5A,B illustrate the selections of 0.5 nm AL lipids of different types surrounding the representative replicates of membrane-bound AA WT-K18 and AA MBD-K18 on CO-raft. Note that only the lipids in one leaflet of the lipid bilayer were selected in both cases. In general, the acyl chain order parameter of the acyl chains of any diacyl chain lipid, DPPC, DLPC, GM1, or POPS, increased progressively, reached a peak between carbon numbers 4 and 8, and declined steadily as the lipid carbon number increased. Note that the chain carbon number starts from 1 (near the polar lipid headgroup) and ends at the terminal methyl carbon at the end of the chain. To improve the statistics of order parameter calculations, the order parameters from both *sn*-1 and *sn*-2 chains of the diacyl chain lipids were combined.

For the CO-raft, the acyl chain orders of DPPC in the AL lipids surrounding the AA WT-K18 tetramer were significantly lower than those surrounding the MBD-K18 tetramer for carbon numbers 1–10 (Figure 5C). Yet, no significant difference in the order parameters of the AL lipids for WT-K18 vs. MBD-K18 was observed for DLPC across all carbon numbers (Figure 5D). As controls, the acyl chain orders of either DPPC or DLPC in the non-AL lipids, nAL, or lipids outside the AL lipid shells, for the AA WT-K18 were identical to that for the AA MBD-K18. The results suggest that the AA WT-K18 perturbed the DPPC and the DLPC chains’ order more than those of the AA MBD-K18 at the top half of the acyl chain region for the CO-raft system. The results indicate that WT-K18, compared to MBD-K18, significantly perturbed the saturated lipids, DPPC, upon binding to the CO-raft.

For the GM-raft, the DPPC acyl chain order of the AL lipids surrounding the AA WT-K18 was slightly lower than those surrounding the AA MBD-K18 for carbon numbers 1–6 (Figure 5E). However, for DLPC, a significantly lower order parameter of the AL lipids surrounding AA WT-K18 than those surrounding AA-MBD-K18 at low carbon numbers 0–4 was detected. However, the trend was reversed at high carbon numbers 14–16 near the end of the acyl chain. The GM1 acyl chain order of the AL lipids surrounding the AA MBD-K18 was slightly lower than those surrounding the AA WT-K18 at large carbon numbers 14–16. For the nAL lipids, the order parameters of DPPC and DLPC were identical for both AA WT-K18 or AA MBD-K18. However, in the presence of AA WT-K18, the GM1 chain orders of the nAL lipids were very similar to those of the AL lipids. In contrast, in the presence of AA MBD-K18, the GM1 chain orders in the nAL lipids were higher than those of the AL and nAL lipids in the presence of AA WT-K18. The above observations indicate that AA-WT-K18 disrupted the DPPC and DLPC chain orders of the AL lipids more than AA-MBD-K18. The GM1 chain order disruptions induced by AA WT-K18 and AA MBD-K18 are similar in the AL lipids. However, the disruptive effect by AA WT-K18 extended to the nAL lipids. In other words, the AA WT-K18 disrupted the GM1 chain orders to the entire GM1 lipid population (AL and nAL), AA MBD-K18 exerted a localized effect at the AL lipid region only. The results indicate that WT-K18 on the GM-raft significantly disordered the acyl chains of nearest DLPC lipids and all the GM-lipids upon binding to the GM1-cluster.

For the PS-raft, no significant difference in the DPPC order parameters of the AL or nAL lipids surrounding the AA WT-K18 and those surrounding the AA MBD-K18 was found. However, the DLPC order parameters of the AL lipids surrounding the AA WT-K18 were slightly lower than those surrounding the AA MBD-K18 at carbon numbers 10–12. The trend was reversed for the POPS order parameters. Again, for the nAL lipids, the order parameters of all nAL lipids surrounding the AA WT-K18 were identical to those surrounding the AA MBD-K18. The lack of significant perturbation of lipid chain order in POPS by both WT-K18 and MBD-K18 suggests that the interactions of protein with the lipids in PS-raft mainly affect the headgroup region with minimal effects at the acyl chains below the headgroups of the lipids.

### 3.6. Protein Folding on Raft Surfaces

**Figure 6:**
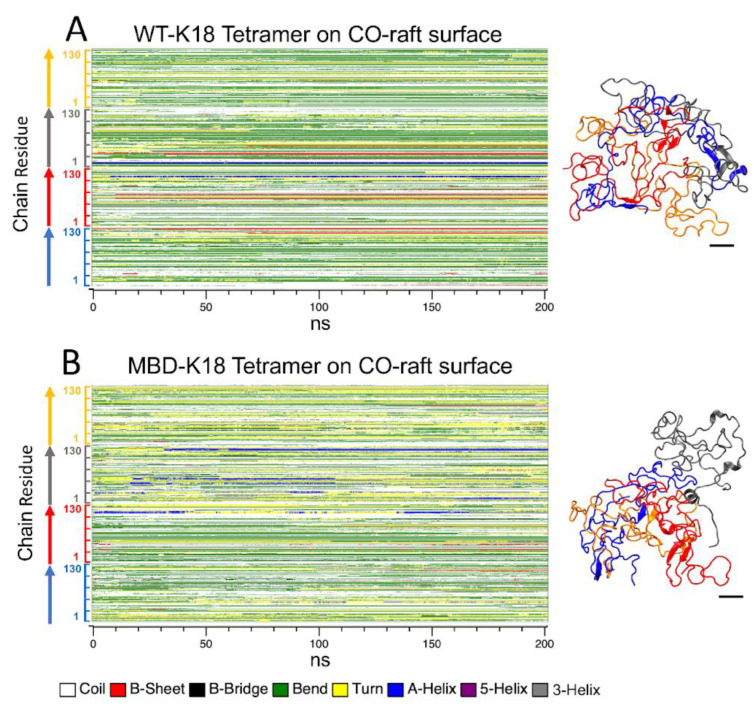
Membrane-templated protein folding of K18 tetramer on CO-raft surfaces. The secondary structures of atomistic WT-K18 (**A**) and MBD-K18 (**B**) represented by DSSP plots in which the secondary structure of each residue was calculated as a function of time of simulation (0 to 200 ns). The structures were also visualized using the VMD program. Each chain is color coded, chain A in blue, chain B in red, chain C in gray, and chain D in orange. The color codes for the secondary structures are also given. The scale bar indicates 10 angstroms.

The protein secondary structures of membrane-bound AA WT-K18 and AA MBD-K18 tetramers on all three different raft surfaces have been calculated using the DSSP (see Materials and Methods) as shown in Supplementary Materials. Within the 200 ns all-atom MD simulations, evidence of transitions from non-hydrogen bonded, disordered structures—such as turn or coil—to hydrogen-bonded, ordered structures—such as bend, helix, or beta—were found for certain regions of the protein. Most of the appearances of the ordered structures, particularly beta-sheet or beta-bridge, are transient. Interestingly, certain replicates, e.g., one replicate of WT-K18 and one replicate of MBD-K18, showed significant beta-sheet formation as shown in Figure 6. In these two replicates, three out of four chains participated in beta-sheet formation. More beta-sheet structures (red strips in the DSSP plot) were detected in WT-K18 than in MBD-K18. The structures and locations of these beta-sheets at the end of the 200 ns-simulation were visualized by VMD (see Materials and Methods), which uses a slightly different secondary structure calculation algorithm than DSSP. Here, 10 beta-sheets in WT-K18 but only 6 beta-sheets in MBD-K18 were detected at 200 ns by VMD. Both inter-chain and intra-chain beta-sheets were found in both WT-K18 and MBD-K18. Therefore, our results indicated that some of our K18 oligomers underwent raft surface-induced folding, i.e., from a disordered state in solution to a partially ordered state in the membrane-bound state, for some of our oligomers within 200 ns of AA simulations in this pilot study. Longer simulation times will be needed to detect more stable beta-sheet structures on the raft surfaces.

## 4. Discussion

Binding kinetics and energetics of Tau-K18 oligomers on various membrane domain models were systematically investigated. Among all three co-existing domain types, Lo, Ld, and Lod, all Tau-K18 oligomers bound to the Lod as demonstrated by the *mindist* vs. time and composition of the annular lipids surrounding the membrane-bound proteins. This Lod domain preference of Tau-K18 binding agrees well with our previous studies on highly ordered beta-amyloid fibrils Aβ_17-42_ [16] and the highly disordered full length Aβ_1-42_ oligomers [17]. Our results, therefore, suggest that the Lod domains are common membrane nanodomain regions for both disordered amyloidogenic proteins, such as beta-amyloid and tau, to bind to. Interestingly, apart from amyloidogenic proteins, another class of membrane-active protein, e.g., HIV-gp42 fusion peptide, has been shown to bind to these Lod domains. The line tension created at the domain boundaries due to the thickness mismatch between the thicker Lo domain and the thinner Ld domain has been proposed to be the major molecular mechanism driving membrane active protein to Lod domain [35,36]. More computational and experimental studies are needed to explore the biophysical mechanisms of protein binding to those self-assembling and dynamic Lod membrane domains.

For the asymmetrically distributed GM-raft containing co-existing GM1-clusters within the Lo, Ld, and Lod domains, or 4 lipid domains on one leaflet, and 3 lipid domains (Lo, Ld, and Lod) on the “opposite” leaflet, tau proteins of all sizes bind exclusively to the GM1-clusters. This exclusive binding property of all our model tau proteins to the GM1-clusters indicates that disordered tau proteins of different sizes have the strong propensity to interact with the GM1-lipids, which are found exclusively on the outer surface of the neuronal membranes [37]. It is well established that initial misfolded and self-aggregated tau proteins are in the cytosol of the neurons [4,6] of the brain. Here, our observations of disordered tau proteins interacting strongly with GM1 lipids, a major biomarker [38], of the outside leaflet of the plasma membranes of neurons, lead us to conclude that GM1 could be a major target of disordered tau oligomers once they are able to migrate and escape from the cytosol of the neurons. Note that a recent work suggests that tau proteins do exit the neurons and translocate to other areas of the brain, including the space between the synaptically associated neurons [5]. In addition, our previous studies on ordered Aβ_17-42_ fibrils and disordered Aβ_1-42_ oligomers also revealed strong binding of amyloid protein aggregates to GM1-clusters [16,17]. It is believed that the presence of highly hydrophilic and disordered carbohydrate groups of the GM1 can provide strong binding sites to the hydrophilic residues of the disordered tau protein. The detailed molecular mechanism of carbohydrate-amino acid residue interaction that stabilizes the tight-binding of GM1 to disordered tau protein remains to be explored computationally and experimentally.

For the asymmetrically distributed PS-raft containing co-existing PS-clusters within the Lo, Ld and Lod domains, or again 4 lipid domains on one leaflet, and 3 lipid domains (Lo, Ld, and Lod) on the “opposite leaflet”, tau tetramer bound exclusively to the PS-clusters on one leaflet. By reducing the charge of Tau-K18 from +10e to +7e, and further decreasing the hydrophobicity of tau, i.e., the mutation from WT-K18 to MBD-K18, a significant decrease in the protein-POPS binding energy as well as a significant difference in the protein-residue minimum distance (*mindist*) at the mutation region I308E were evident. The results suggest that electrostatic interaction between the cationic K18 and negatively charged PS is a major membrane binding mechanism of Tau-K18 with PS-containing membrane domains, exclusively found in the inner leaflet of neuronal membranes. Our observations agree with previous experimental work on the preferred binding of WT-K18 over MBD-K18 on PS containing liposomes, and hence validate our computational approach of investigating disordered tau oligomer interactions with raft membranes.

Other than identifying the membrane domain targets of Tau-K18, we have also attempted to quantitate the extent of membrane structural disruption, or membrane damage, and the associated membrane-induced beta-sheet formation in the presence of the membrane-bound Tau-K18 oligomers. Membrane damage and beta-sheet formation are considered to be the major factors associated with the interaction of tau aggregates with lipid membranes and contribute significantly to the early molecular events of tauopathies that initiate the onset of Alzheimer’s [1,4,6]. Substantial disruptions of lipid structural packing, as quantified by a significant decrease in the orientational order parameter of annular lipids surrounding the membrane-bound tau oligomers, were evident. Interestingly, WT-K18 disrupted the annular lipids more than MBD-K18, particularly the saturated DPPC in all lipid rafts. The observation of a stronger order disruption of unsaturated DLPC by WT-K18 than by MBD-K18 in GM-raft suggests that WT-K18 was able to perturb the unsaturated lipid more effectively than MBD-K18. Since the orientational order of lipid acyl chains are important to maintain the physiological functions of membranes, a large disruption in the lipid order will create detrimental effects on the structure–function relationship of membranes, inducing tauopathies associated with membranes [2].

Although our tau protein is a relatively large amyloid aggregate when compared to, say, Aβ_1-42_ (with tetramers of 520 residues vs. 168 residues, respectively), significant but localized beta-sheet formations in the membrane-bound tau aggregates were evident in some of our simulation replicates. For the case of the tau tetramer on the CO-raft, small interchain and intrachain beta-sheet formations were found, and WT-K18 appeared to produce and sustain more beta-sheets than MBD-K18. Certainly, much longer simulation time and advanced sampling techniques are required to further explore the role of membrane domains on membrane-bound beta-sheet formations. Our preliminary results in this study indicate that membrane-assisted beta-sheet formation, starting from a disordered solution state to the membrane-bound state, can be detected for relatively large amyloid protein aggregates like Tau-K18. Experimental observations of beta-sheet formation on the lipid membrane surface, particularly anionic membrane surface, of Tau-K18 has recently been reported [12]. Our atomistic simulation work provides computational evidence that interchain and intrachain beta-sheet formations are not only possible, but likely to occur on anionic membrane surfaces.

The formation of small beta-sheets on membrane-bound amyloid aggregates may represent the early events of creating more membrane-disruptive structures associated with the pore, carpeting and detergent-like models [2]. The higher rate of beta sheet formation in WT-K18 over MBD-K18 suggests that electrostatic interactions play a key role in membrane-templated protein folding [12]. Furthermore, these small beta-sheet structures may act as seeds to attract or recruit more amyloid proteins from solution or adjacent membrane-bound amyloid proteins to create even more membrane-disruptive aggregates that further damage the membranes [4]. Therefore, the observations of small beta-sheets in this pilot study provide the base for future experimental or computational work investigating the nucleation processes of tau on membrane domains [4,6].

In this study, we have used multiscale molecular dynamics simulations to investigate early tau binding events to three structurally distinctive model raft membranes on the microsecond time scale and with both coarse-grained (CG) and atomistic (AA) spatial resolutions. CG simulations allow us to explore protein-lipid binding events up to tens of microseconds, while subsequent AA simulations provide protein-folding and detailed nonbonded interactions, such as electrostatic and van der Waals, that CG simulations do not. Due to the large number of atoms, partial charge distributions of molecules, and a lack of phase sampling capability of conventional AA simulations, limited protein-lipid conformational sampling around the pre-established protein-lipid contact regions can only be explored within a few hundred nanoseconds. Our multiscale simulations represent a useful approach to investigate protein-lipid binding events and protein-folding on surfaces upon binding associated with tau-membrane interactions. Note that this multiscale simulation approach has successfully been applied to other systems, such polymer-ion complex and protein in solution [39,40,41].

## 5. Conclusions

Using multiscale molecular dynamic simulations (CG-MD and AA-MD), the binding behaviors of WT-K18 and MBD-K18 oligomers onto phase-separated lipid domains, with and without anionic biomarkers of the inner and outer leaflet of the neuron, were explored. By using models mimicking different domains of realistic neuronal membranes and multiscale simulation, we were able to holistically investigate binding events of disordered tau aggregates on lipid rafts, as well as high-resolution protein folding and protein-lipid conformation. Electrostatic interactions between Tau-K18 and lipid domains indicate the preferential binding regions of tau oligomers, prioritizing in the order of GM1 clusters (outer membrane leaflet’s biomarker), then PS cluster (inner membrane leaflet’s biomarker), and finally, the mixed Lod domain. Additionally, detailed membrane-order analyses in atomistic resolution suggest significant membrane damage caused by both WT-K18 and MBD-K18 to CO-raft and GM-raft, but not PS-raft, suggesting the membrane disruptive of tau oligomers, especially WT-K18 oligomers, once they delocalize from the inside of a neuron. Beta-sheet motifs in membrane templated-protein folding were also evident, where WT-K18 had higher propensity for beta sheet formations than MBD-K18, suggesting the toxicity of disordered tau oligomers and potential seeding effect for further membrane damage. Overall, results indicated that the three point mutations (V287E, I308E, V318E) in MBD-K18 successfully decreased membrane binding and disruption on neuronal surfaces, but still significantly affected membrane lipid orientations, especially when relocated to outer neuronal leaflets containing GM1 clusters. The technique of multiscale MD simulation and analysis provides a new approach to understand the insights of tauopathic membrane-associated mechanisms and damage.

## Figures and Tables

**Table 1:** Summary of lipid binding times of tau oligomers and compositions of 0.5 nm-annular lipids surrounding the membrane-bound tau oligomers of different aggregation sizes, monomer (*n* = 1), dimer (*n* = 2) and tetramer (*n* = 4), on different raft surfaces.

Raft	Tau	*n*	Binding Time (μs)			CHOL% *	DPPC% *	DLPC% *	GM1 or POPS% *	Number of Lipids *
CO	CG-WT	1	1.72	0.89	0.17	29 ± 1	47 ± 1	24 ± 2		15 ± 1
		2	20.00	16.94	14.18	27 ± 7	50 ± 13	23 ± 1		15 ± 2
		4	7.63	0.93	0.07	29 ± 3	46 ± 4	25 ± 3		20 ± 1
	CG-MBD	1	7.15	1.00	6.42	25 ± 3	40 ± 6	35 ± 4		12 ± 1
		2	3.27	11.30	7.88	27 ± 4	48 ± 4	24 ± 5		13 ± 1
		4	0.08	0.49	2.78	27 ± 3	44 ± 5	29 ± 4		20 ± 1
GM	CG-WT	1	2.85	1.07	0.09	19 ± 4	32 ± 4	14 ± 2	35 ± 2	66 ± 2
		2	0.15	0.11	0.08	23 ± 2	35 ± 2	15 ± 2	27 ± 2	86 ± 3
		4	0.58	0.30	0.23	22 ± 2	33 ± 2	15 ± 3	30 ± 2	115 ± 2
	CG-MBD	1	0.15	0.67	0.57	10 ± 2	14 ± 3	10 ± 1	66 ± 10	30 ± 3
		2	1.70	0.51	0.64	16 ± 2	27 ± 3	11 ± 2	46 ± 6	27 ± 2
		4	0.17	0.48	1.62	12 ± 1	21 ± 4	14 ± 2	53 ± 8	48 ± 4
PS	CG-WT	1	1.04	0.57	0.92	28 ± 3	23 ± 3	17 ± 2	31 ± 2	19 ± 1
		2	1.08	0.20	1.87	30 ± 2	25 ± 2	12 ± 2	34 ± 4	27 ± 1
		4	0.87	2.33	4.07	30 ± 5	22 ± 4	12 ± 2	36 ± 5	44 ± 4
	CG-MBD	1	0.04	5.41	4.90	28 ± 2	23 ± 2	18 ± 3	32 ± 1	18 ± 1
		2	5.60	0.28	0.55	28 ± 1	23 ± 2	16 ± 2	33 ± 3	24 ± 1
		4	3.22	5.55	1.40	31 ± 3	24 ± 2	10 ± 1	36 ± 3	35 ± 2
CO	AA-WT	4				26 ± 4	53 ± 9	21 ± 6		39 ± 5
	AA-MBD	4				25 ± 7	56 ± 6	18 ± 1		37 ± 3
GM	AA-WT	4				16 ± 2	38 ± 2	16 ± 6	30 ± 2	66 ± 5
	AA-MBD	4				17 ± 2	32 ± 8	24 ± 2	27 ± 2	65 ± 7
PS	AA-WT	4				29 ± 6	25 ± 3	16 ± 2	30 ± 2	75 ± 7
	AA-MBD	4				28 ± 5	24 ± 5	19 ± 5	29 ± 2	64 ± 6

* The uncertainties are standard errors of the means over the last 5 µs (CG) or 50 ns (AA) of simulations and across independent replicates.

## Data Availability

Not applicable.

## Supplementary Materials

The following supporting information can be downloaded at: https://www.mdpi.com/article/10.3390/membranes12111098/s1. Figure SA1. Sequence alignments of WT-K18, MBD-K18 and Fibril Core of K18. Figure SB1. Contact maps of monomeric and tetrameric CG WT-K18 in solution. Figure SB2. Contact maps of monomeric and tetrameric CG WT-K18 on CO-raft surfaces. Figure SC1. Aggregation kinetics of CG WT-K18 oligomers. Figure SC2. Periodic images of lipid rafts. Figure SC3. Binding of CG WT-K18 dimer to CO-raft and GM-raft. Figure SC4. Binding of WT-K18 dimer from solution to membrane-bound states for the PS-raft. Figure SC5. Compositions of Lo, Ld and Lod domain in lipid rafts. Figure SD1. Minimum distance analysis of K18 tetramer binding to lipid rafts. Figure SD2. *Mindist* spectra of K18 oligomers on CO-raft surface. Figure SD3. *Mindist* spectra of CG WT-K18 and CG MBD-K18 on GMCO-raft surface. Figure SD4. *Mindist* spectra of CG WT-K18 and CG MBD-K18 on PS-raft surface. Figure SD5. *Mindist* spectra of CG tetramer K-18 (A,B) andAA Tetramer K-18 (C,D) on CO-raft surface. Figure SD6. *Mindist* spectra of CG tetramer K-18 (A,B) andAA Tetramer K-18 (C,D) on GM-raft surface. Figure SD7. *Mindist* spectra of CG tetramer K-18 (A,B) andAA Tetramer K-18 (C,D) on PS-raft surface. Figure SE1. Number of lipids in the 1.2 nm annular lipid shell. Figure SE2. Normalized protein-lipid binding. Figure SF1. DSSP plots of WT-K18 and MBD-K18 on CO-raft. Figure SF2. DSSP plots of WT-K18 and MBD-K18 on GM-raft. Figure SF3. DSSP of WT-K18 and MBD-K18 on PS-raft.
