# Supplementary material for: Exploring Membrane Binding Targets of Disordered Human Tau Aggregates on Lipid Rafts Using Multiscale Molecular Dynamics Simulations
Source: Membranes (Basel). 2022 Nov 4;12(11):1098. doi: 10.3390/membranes12111098 (PMC9695534; doi:10.3390/membranes12111098)
Supplement: Supplementary file 1 [file membranes-12-01098-s001.zip › membranes-1972452-supplementary.pdf]

membranes

Article

Supplementary Information (SI)—Exploring Membrane Binding Targets of Disordered Human Tau Aggregates on Lipid Rafts Using Multiscale Molecular Dynamics Simulations

Kwan H. Cheng [1,2,*], Angela Graf [2], Amber Lewis [1], Thuong Pham [2], and Aakriti Acharya [2]

[1] Neuroscience Department, Trinity University, San Antonio, TX 78212, USA
[2] Physics Department, Trinity University, San Antonio, TX 78212, USA
* Correspondence: kcheng1@trinity.edu; Tel.: +1-210-999-8469

Citation: Cheng, K.H.; Graf, A.; Lewis, A.; Pham, T.; Acharya, A. Supplementary Information (SI)—Exploring Membrane Binding Targets of Disordered Human Tau Aggregates on Lipid Rafts Using Multiscale Molecular Dynamics Simulations. *Membranes* **2022**, *12*, 1098. https://doi.org/10.3390/membranes12111098

Academic Editor: Masanao Kinoshita

Received: 30 September 2022
Accepted: 1 November 2022
Published: 4 November 2022

Publisher's Note: MDPI stays neutral with regard to jurisdictional claims in published maps and institutional affiliations.

Copyright: © 2022 by the authors. Licensee MDPI, Basel, Switzerland. This article is an open access article distributed under the terms and conditions of the Creative Commons Attribution (CC BY) license (https://creativecommons.org/licenses/by/4.0/).

SI - A

Sequence alignments of WT-K18, MBD-K18 and K18 fibril core are shown in Fig. A1. WT-K18 and MBD-K18 contain all four R1 to R4 repeats, while the fibril core contains only R3 and R4. In all sequences, the cationic and anionic residues are marked in blue and red colors, respectively, while the hydrophobic residues are underlined. The common PGGG pattern in the repeats is also shown.

A. TAU-WT-K18

^{243}LQTAPVPMPDLK-NVKSKIGSTENLKHQPGGGK^{274} (R1)
^{275}VQIINKKLDLS-NVQSKCGSKDNIKHVPGGGS^{305} (R2)
^{306}VQIVYKPVDLS-KVTSKCGSLGNIHHKPGGGQ^{336} (R3)
^{337}VEVKSEKLDFKDRVQSKIGSLDNITHVPGGGN^{368} (R4)
^{369}KKIE^{372}

B. TAU-MBD-K18 (V287E I308E V318E)

^{243}LQTAPVPMPDLK-NVKSKIGSTENLKHQPGGGK^{274} (R1)
^{275}VQIINKKLDLS-NEQSKCGSKDNIKHVPGGGS^{305} (R2)
^{306}VQEVYKPVDLS-KETSKCGSLGNIHHKPGGGQ^{336} (R3)
^{337}VEVKSEKLDFKDRVQSKIGSLDNITHVPGGGN^{368} (R4)
^{369}KKIE^{372}

C. TAU-FC-K18

^{306}VQIVYKPVDLS-KVTSKCGSLGNIHHKPGGGQ^{336} (R3)
^{337}VEVKSEKLDFKDRVQSKIGSLDNITHVPGGGN^{368} (R4)
^{369}KKIETHKLTF^{378}

Figure SA1. Sequence alignments of WT-K18, MBD-K18 and Fibril Core of K18. The R1, R2, R3 and R4 repeats of Tau-WT-K18 (A), Tau-MBD-K18 (B) and Tau fibril core, or Tau-FC-K18, are labeled. The cationic, anionic, and hydrophobic residues are marked as red, blue, and underlined, respectively. The common region of PGGG in each repeat is highlighted in yellow.

SI - B

Protein residue contact maps were used to investigate the time-averaged and standard deviation of intra-chain and inter-chain residue contacts for monomeric and tetrameric WT-K18 in solution (Fig. B1) and on CO-raft surfaces (Fig. B2). Diffused contact regions and large fluctuations were evident for WT-K18 in solution. In contrast, focused contact regions and less fluctuations were found when the protein established binding on raft surfaces. The results indicate a significant reduction of the non-bonded conformational phase space of the protein from the 3D solution state to the 2D planar membrane-bound state.

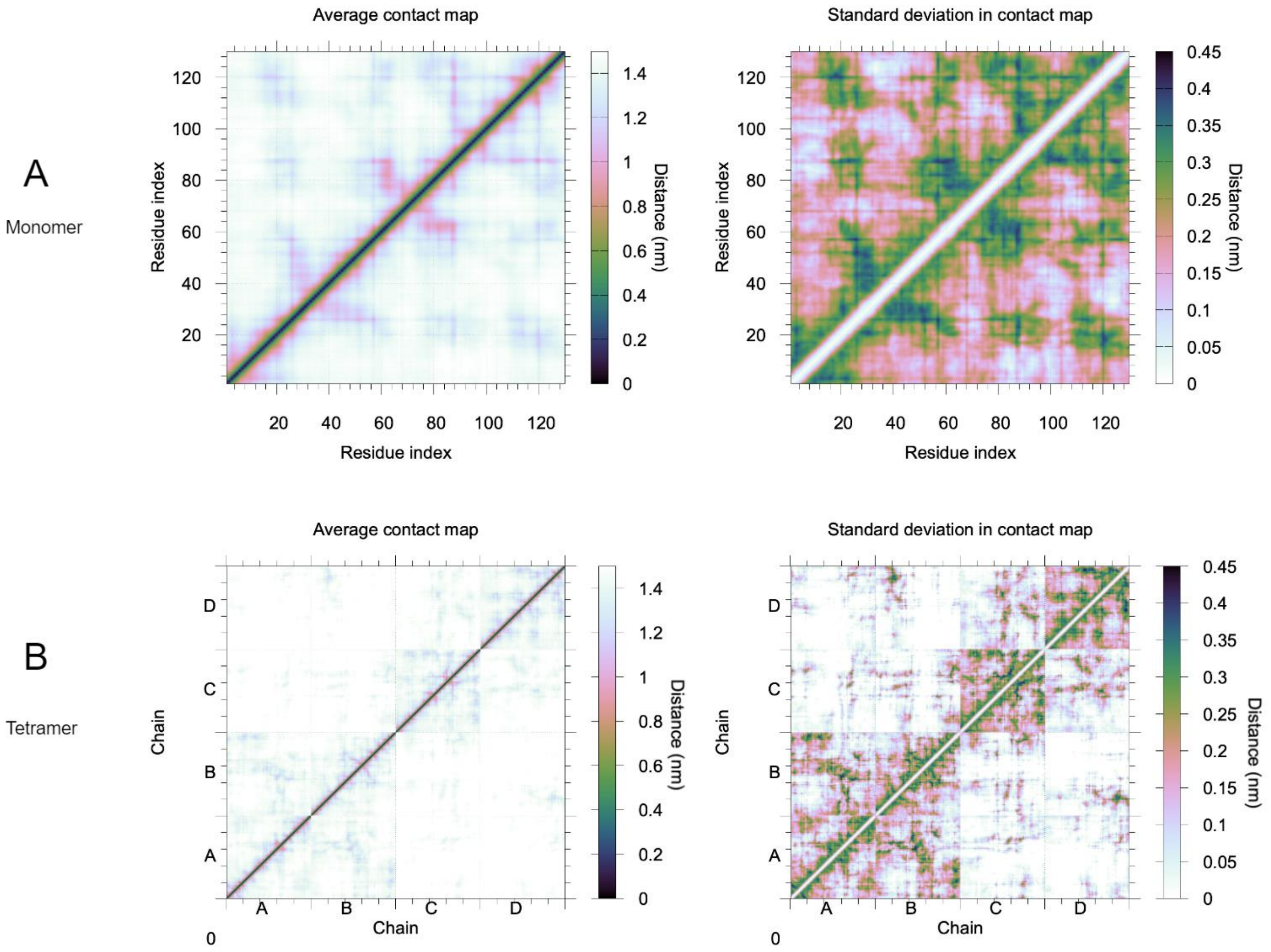

Figure SB1. Contact maps of monomeric and tetrameric CG WT-K18 in solution. (A) The average (left panel) and the standard deviation (right panel) of contact between each amino acid residue in a WT-K18 monomer, over 2.5 μs of simulation. (B) The average (left panel) and the standard deviation (right panel) of contact between each amino acid residue in a WT-K18 tetramer, over 5 μs of simulation.

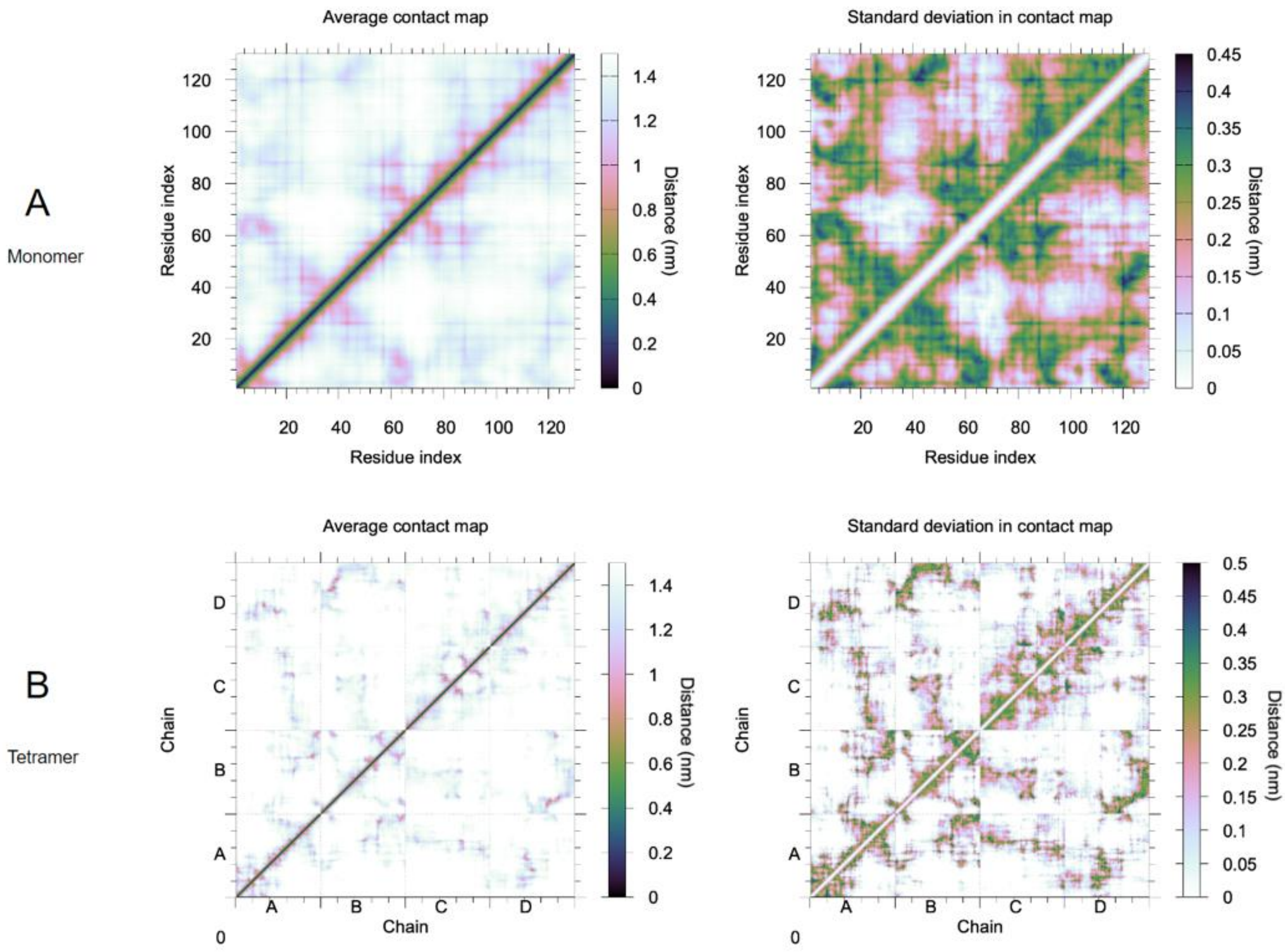

Figure SB2. Contact maps of monomeric and tetrameric CG WT-K18 on CO-raft surfaces. (A) The average (left panel) and the standard deviation (right panel) of contact between each amino acid residue in a WT-K18 monomer. (B) The average (left panel) and the standard deviation (right panel) of contact between each amino acid residue in a WT-K18 tetramer. All were from 10 to 15 μs of simulation.

SI - C

Aggregation kinetics of WT-K18 dimer and tetramer in solution are shown in Fig. C1. The Lo, Ld, Lod and GM1-cluster domains in the absence (Fig. C2) and presence of membrane-bound WT-K18 dimer (Figs. C3 and C4) are given. The PS-clusters are mainly sequestered into the Lo and Lod domains and excluded from the Ld domains. To provide better visualization of the domain arrangement, multiple self-repeated, periodic images are shown in Fig. C2. The lipid compositions of the lipid domains are given in Fig. C5.

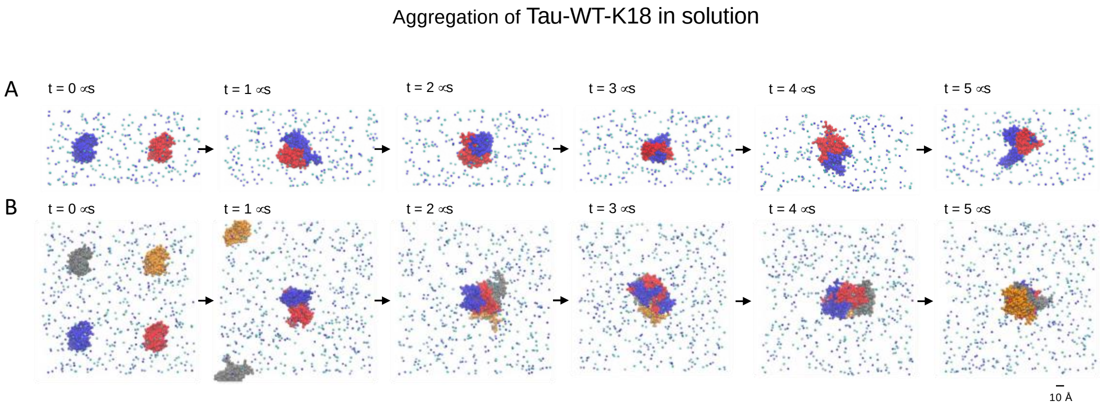

Figure SC1. Aggregation kinetics of CG WT-K18 oligomers. The aggregation states of dimer (A) and tetramer (B) of WT-K18 starting from evenly spaced monomers (t = 0 µs) to oligomers of different sizes at different times are shown. The scale bar indicates 10 angstroms. Protein chains are labeled in blue (chain A), red (chain B), gray (chain C) and orange (chain D). The water (blue dot), and ions with dark blue for Na+ and light blue for Cl-, are shown.

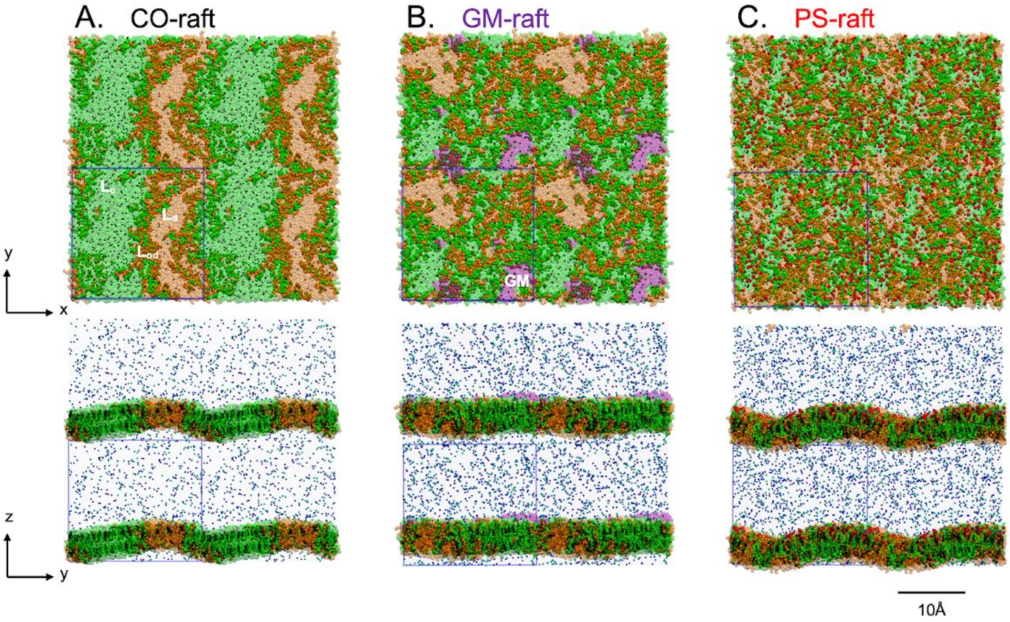

Figure SC2. Periodic images of lipid rafts. Lateral (x-y) and transverse (y-z) periodic images of CO-raft (A), GM-raft (B) and PS-raft (C) after 10 µs of CG MD simulation in the absence of externally added protein. All simulations were performed in the presence of 0.1 M NaCl, 310 K and 1 atmospheric pressure. The lipid types are color coded: DPPC (green), DLPC (orange), CHOL (black), GM1 (magenta) and POPS (red). For the transverse images, waters are in small blue dots and ions are in dark blue (Na+) and light blue (Cl-). The Lo, Ld and Lod domains are shown in light green, light orange and bright green or orange, respectively, for all rafts. The scale bar indicates 10 angstroms.

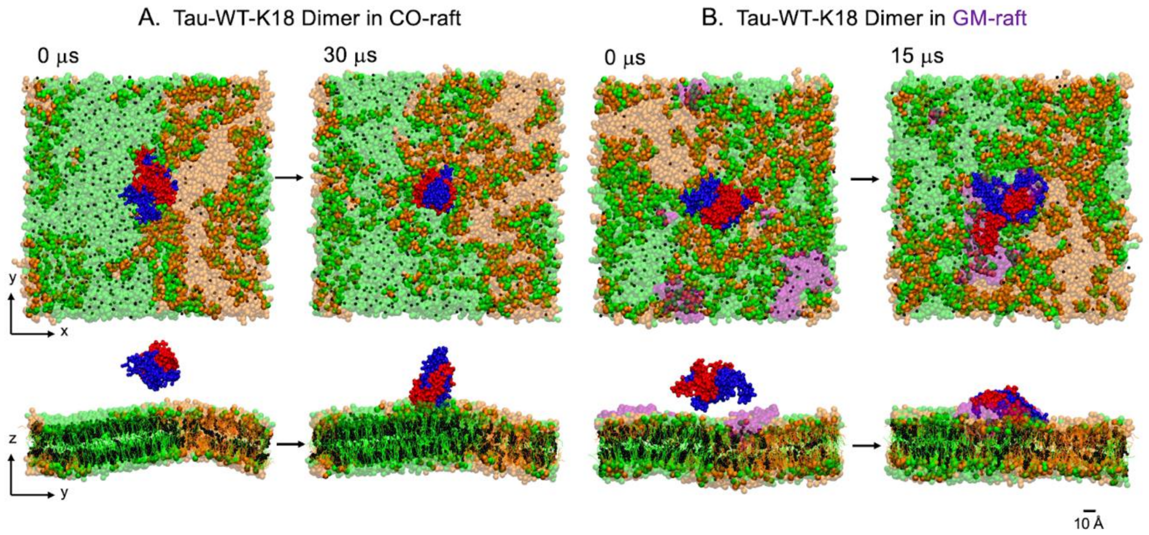

Figure SC3. Binding of CG WT-K18 dimer to CO-raft and GM-raft. Binding of WT-K18 dimer from the solution state (0 μs) to the equilibrated membrane-bounded state for the CO-raft at 30 μs (A) and at 15 μs for the GM-raft (B). The chain A and chain B of the dimer are in blue and red beads, respectively. The scale bar indicates 10 angstroms. For clarity, the water and ions are not shown. See the legend of Fig. C2 for more details of the labeling of membrane domains.

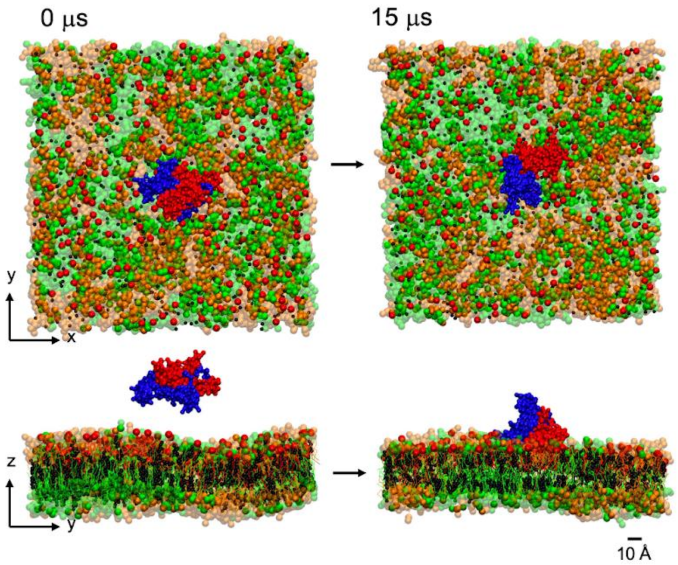

Figure SC4. Binding of WT-K18 dimer from solution to membrane-bound states for the PS-raft. Binding of WT-K18 dimer from the solution state (0 μs) to the equilibrated membrane-bounded state for the PS-raft at 15 μs. The chain A and chain B of the dimer are in blue and red beads, respectively. The scale bar indicates 10 angstroms. For clarity, the water and ions are not shown. See the legend of Fig. C2 for more details of the labeling of membrane domains.

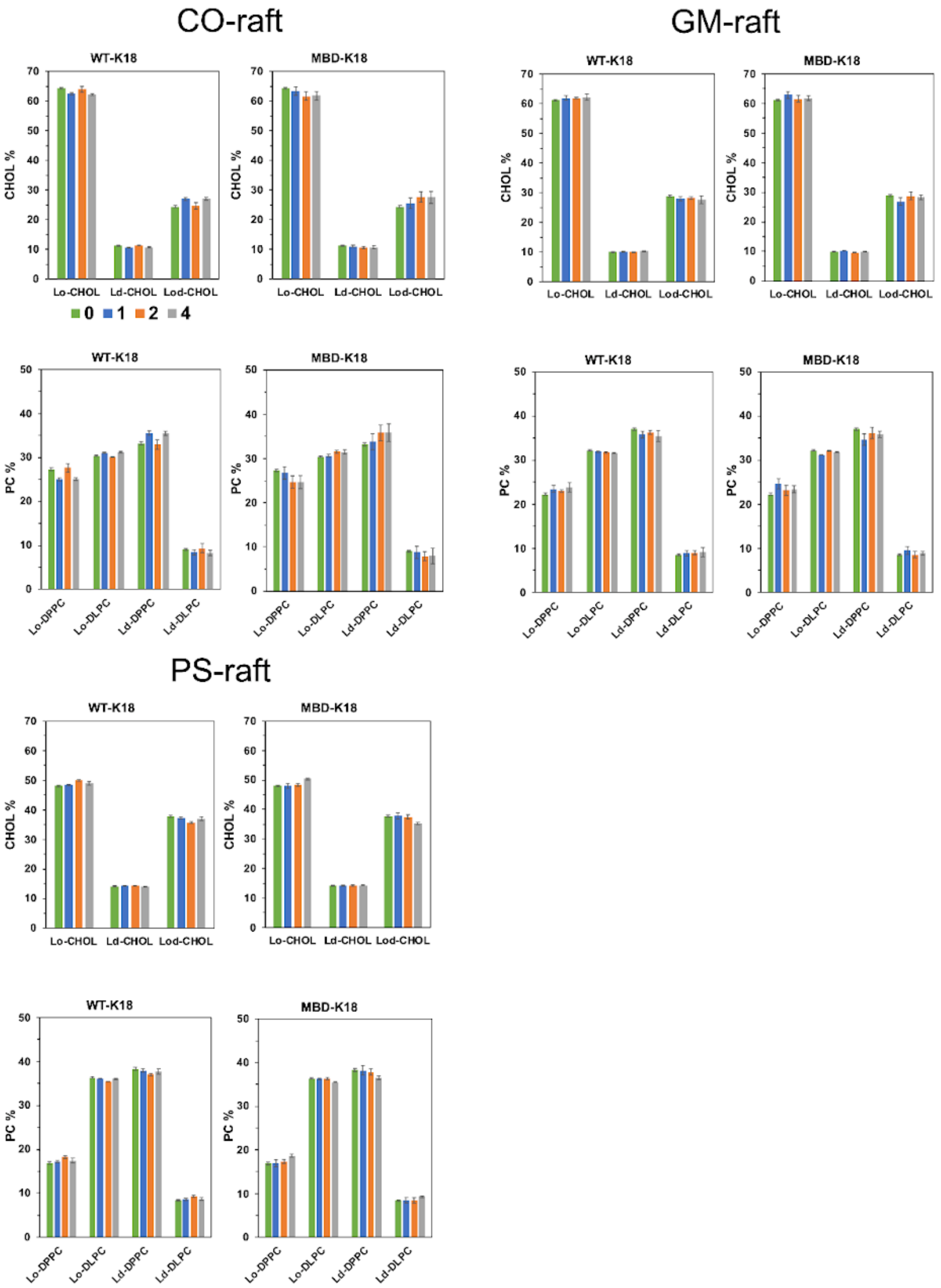

Figure SC5. Compositions of Lo, Ld and Lod domain in lipid rafts. Time- and replicate averaged percentages of DPPC, DLPC and CHOL in Lo, Ld and Lod domains of CO-raft, GM-raft and PS-raft over the last 5 μs and across three independent simulation replicates for each system are given. All three oligomers, monomer (blue), dimer (red) and tetramer (gray), are shown. The control (green) represents the data in the absence of protein. The error bars are standard errors of the means.

SI - D

Representative minimum distance analysis of WT-K18 and MBD-K18 in lipid rafts at both CG and AA resolutions are shown in Fig. D1. Similar to dimers in Fig. 3, stable membrane-bound tetramers were found for CG WT-K18 and MBD-K18 tetramers in all lipid rafts. After the CG-to-AA resolution transformation of the last frame of the CG-simulation, a stable membrane-bound state of each AA tetramer was detected for both WT-K18 and MBD-K18 as shown in the *mindist* vs. time and number of contacts vs. time curves.

A *mindist* spectrum allows us to identify specific protein residue patterns, or membrane binding domains, where the K18 oligomers prefer to attach on different raft surfaces. Here, the values of *mindist* for protein-lipid and protein-water are shown. The *mindist* spectra of CG K18 oligomers in CO-raft (Fig. D2), GM-raft (Fig. D3) and PS-raft (Fig. D4) for WT-K18 (upper panel) and MBD-K18 (mid panel) are given. In addition, the difference spectrum, defined as WT-K18 spectrum subtracted by- MBD-K18 spectrum, for each simulation system is presented (bottom panel). The data points represent time- and replicate-averaged values over the last 5 μs and across all three independent replicates of each simulation system. Hence, a total of 18 *mindist* spectra, representing the monomer, dimer and tetramer of either CG WT-K18 or CG MBD-K18 on three different rafts, are presented. Also, a comparison of the binding pattern of CG Tetramer K-18 and AA Tetramer K-18 on CO-raft (Fig. D5), GM-raft (Fig. D6) and PS-raft (Fig. D7) are shown.

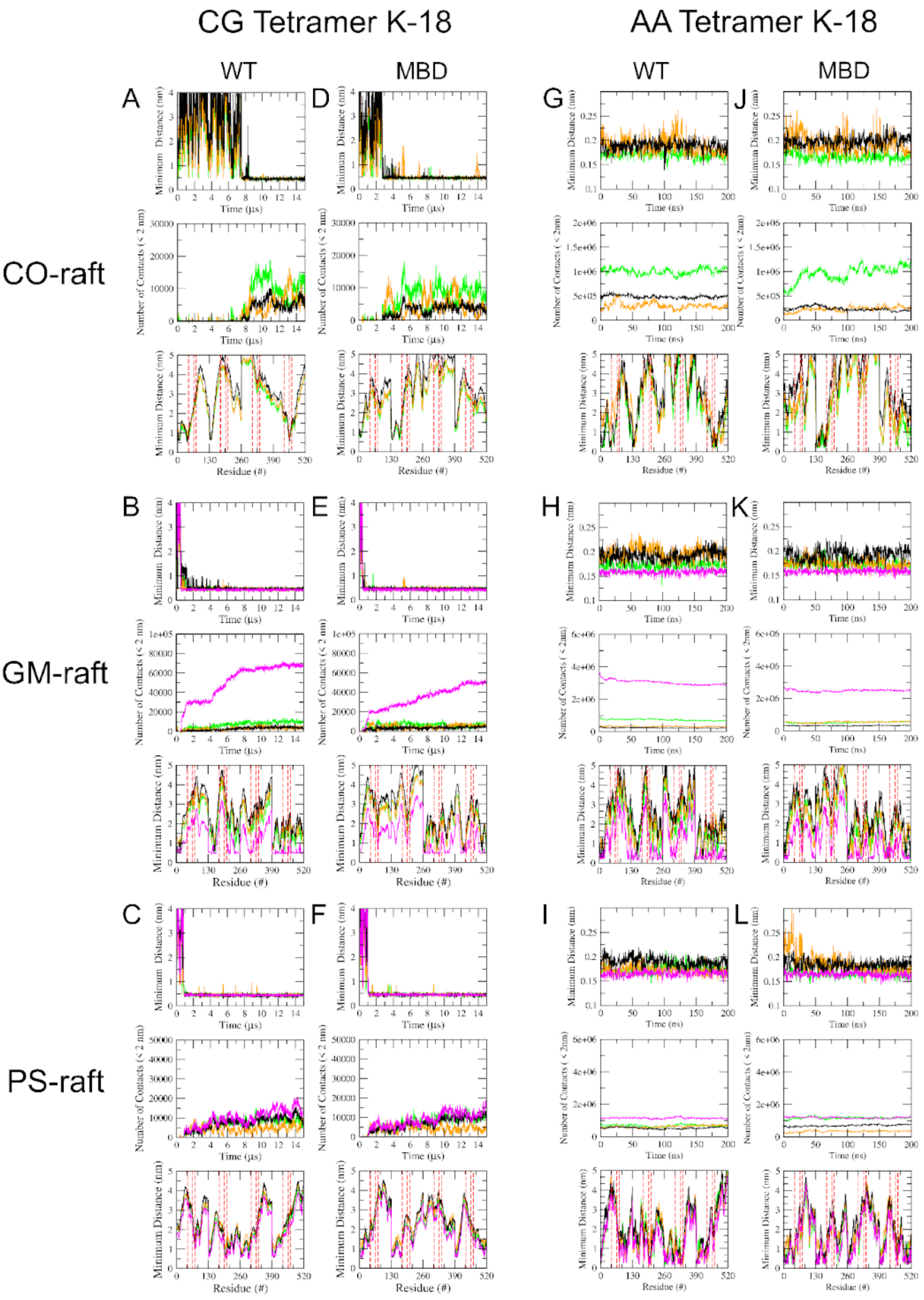

Figure SD1. Minimum distance analysis of K18 tetramer binding to lipid rafts. Minimum distance analysis of K18 tetramer at CG and AA resolutions in CO-raft, GM-raft and PS-raft. See the legend of Fig. 3 for details.

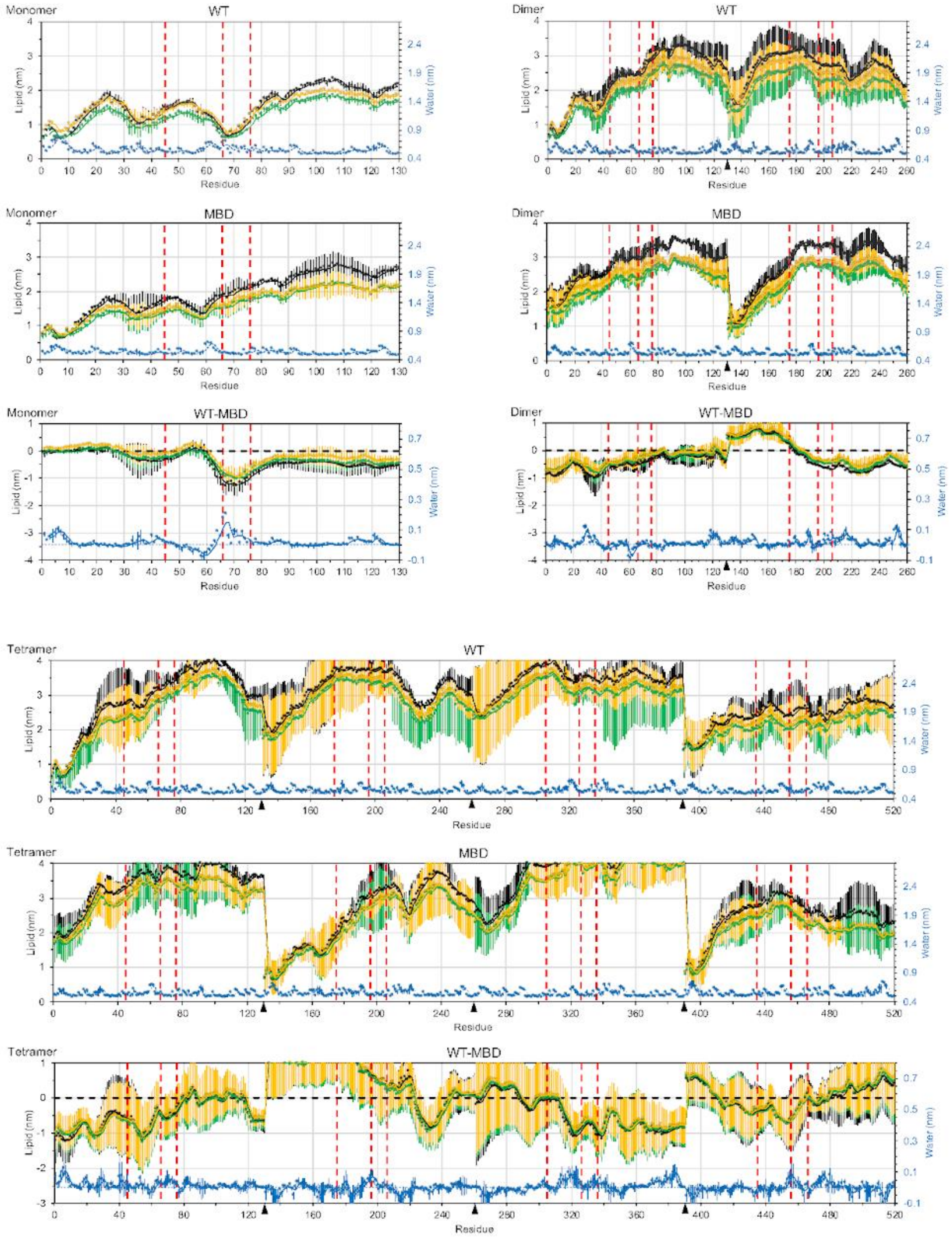

Figure SD2. *Mindist* spectra of K18 oligomers on CO-raft surface. The spectra of CG WT-K18 (WT) and CG MBD-K18 (MBD) are shown in the top two panels, respectively. The bottom panel shows the difference spectrum (= spectrum of WT - spectrum of MBD). The data points represent time- and replicate-averaged values over the last 5 µs and across all three independent replicates of each system. The lipid types are color coded with DPPC in green, DLPC in orange, CHOL in black, water in blue. Three mutation markers (dashed red lines) for each protein chain at residues 45, 66 and 76 of chain A, 175, 196 and 206 of chain B, 305, 326 and 336 of chain C, and 435, 456 and 466 of chain D are given. The black arrow heads on the residue axis identify the N-termini or locations of the starting residues of chain B, (residue 131), chain C (residue 261) and chain D (residue 391). A 5 point-moving average curve was drawn for each spectrum. The error bars represent standard errors of the means.

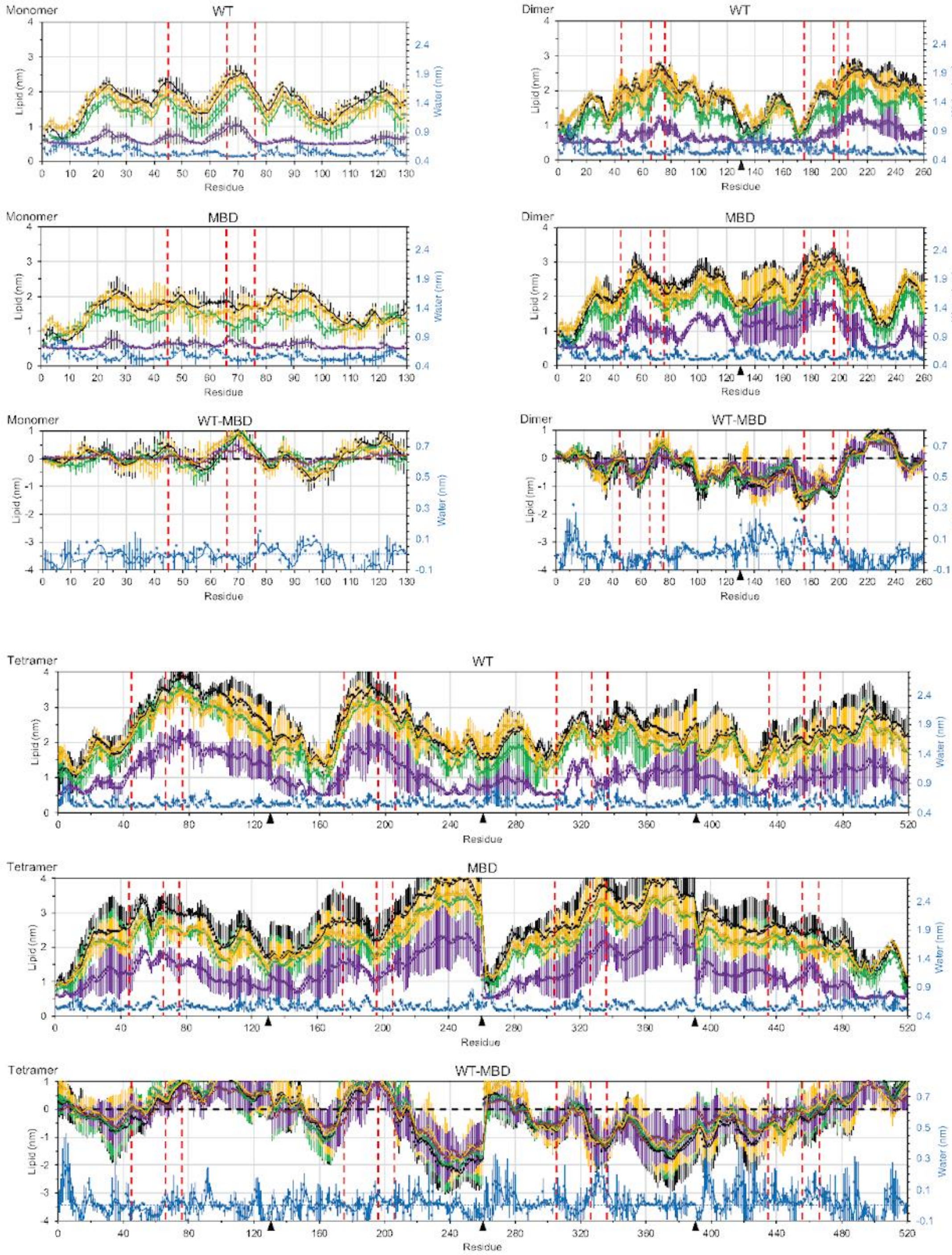

Figure SD3. *Mindist* spectra of CG WT-K18 and CG MBD-K18 on GMCO-raft surface. The color labels of the spectra are identical to those for the CO-raft in Fig. D23, except the presence of the additional GM1 in magenta.

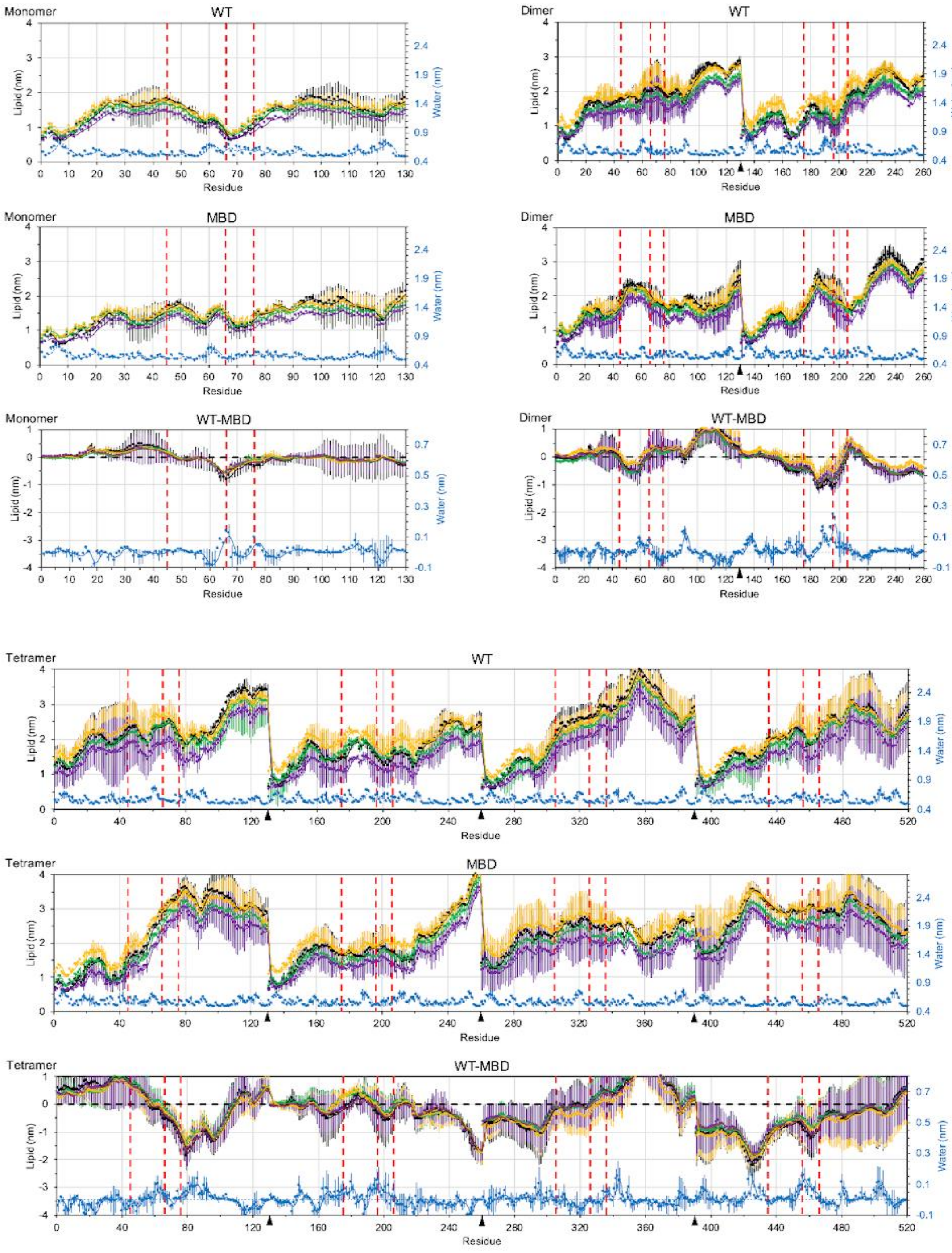

Figure SD4. *Mindist* spectra of CG WT-K18 and CG MBD-K18 on PS-raft surface. The color labels of the spectra are identical to those for the CO-raft in Fig. D23, except the presence of the additional POPS in magenta.

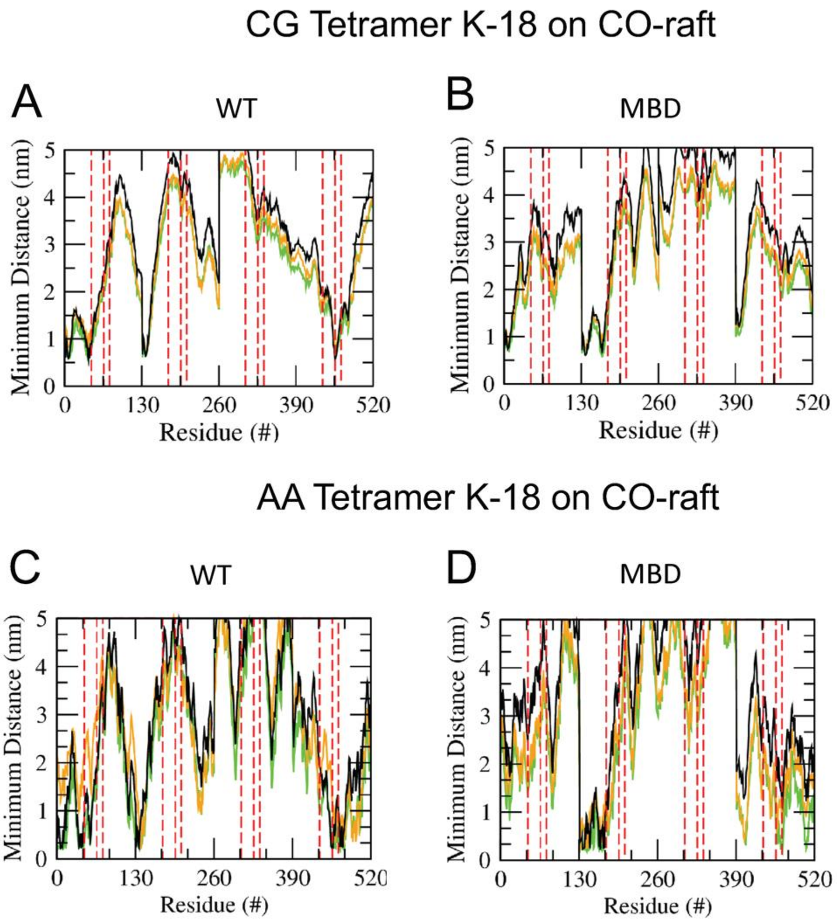

Figure SD5. *Mindist* spectra of CG tetramer K-18 (A, B) andAA Tetramer K-18 (C, D) on CO-raft surface. Both WT-K18 (A, C) and MBD-K18 (B, D) are shown. See the legend of Fig.3 for details.

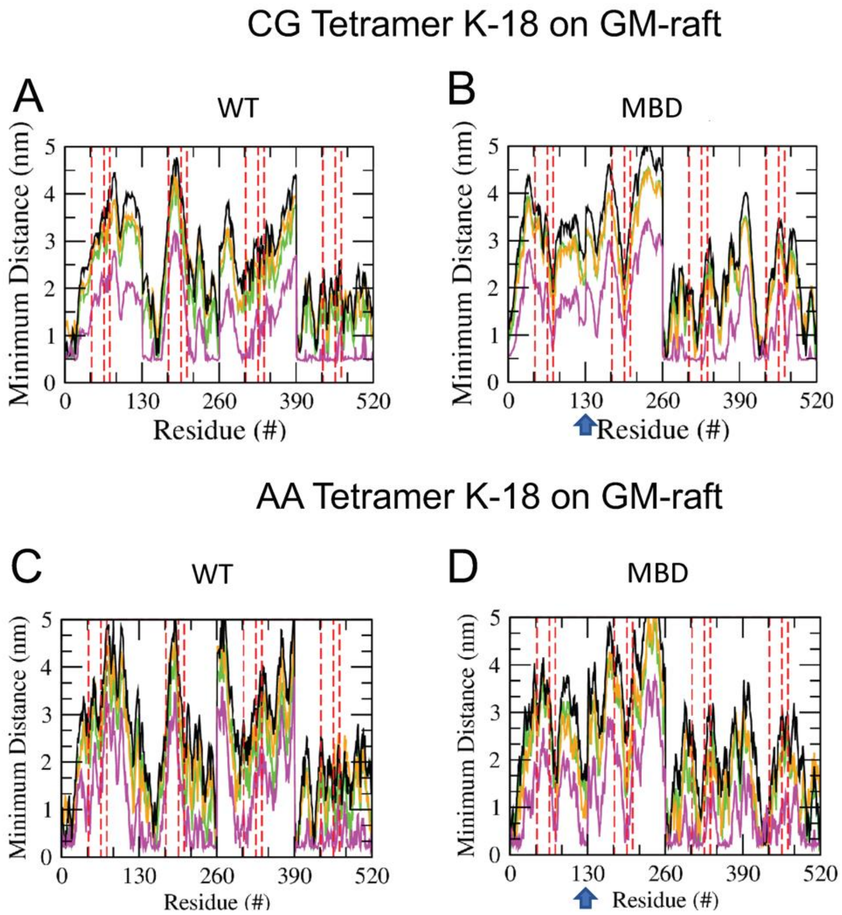

Figure SD6. *Mindist* spectra of CG tetramer K-18 (A, B) andAA Tetramer K-18 (C, D) on GM-raft surface. Both WT-K18 (A, C) and MBD-K18 (B, D) are shown. See the legend of Fig.3 for details.

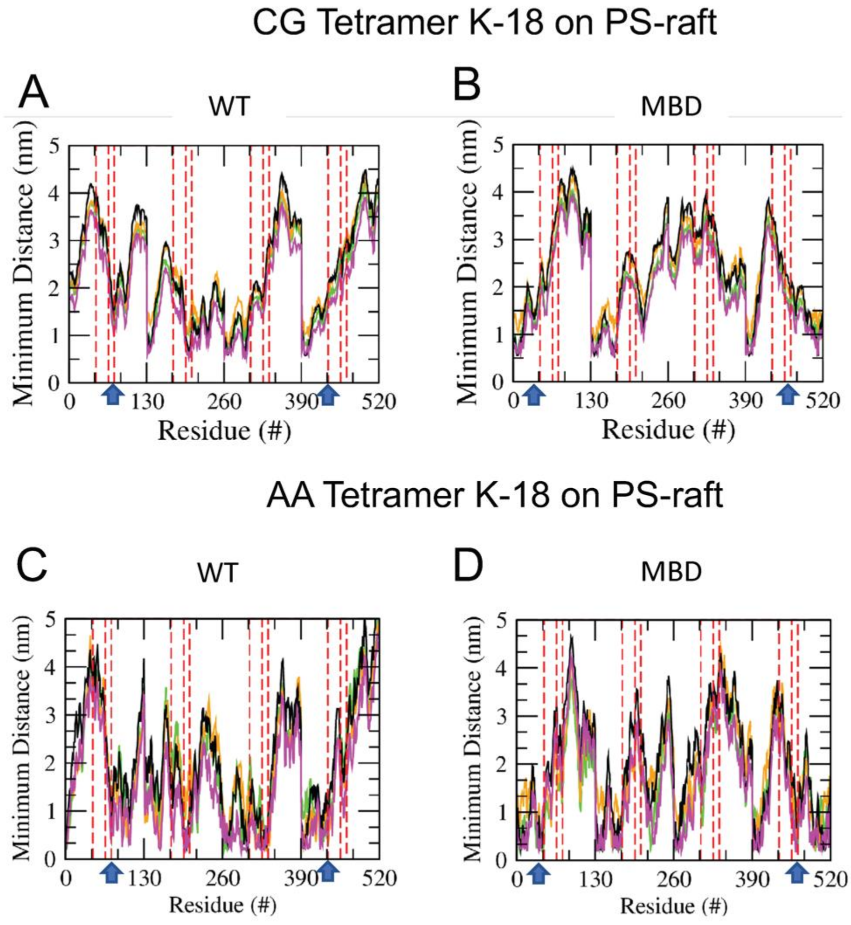

Figure SD7. *Mindist* spectra of CG tetramer K-18 (A, B) andAA Tetramer K-18 (C, D) on PS-raft surface. Both WT-K18 (A, C) and MBD-K18 (B, D) are shown. See the legend of Fig.3 for details.

SI - E

The time- and replicate-averaged number of lipids for each lipid type inside the 1.2 nm annular lipid shell surrounding the membrane-bound WT-K18 and MBD-K18 oligomers over the last 5 μs for CG or 50 ns for AA and across all three independent replicates are shown in Fig. E1. Also shown are the total number of lipids surrounding the oligomers. By dividing the total protein-lipid binding energy in Fig. 4 with the corresponding numbers of lipids shown here, the normalized protein-lipid binding energy for each lipid type and for all lipids were generated and are shown in Fig. E2.

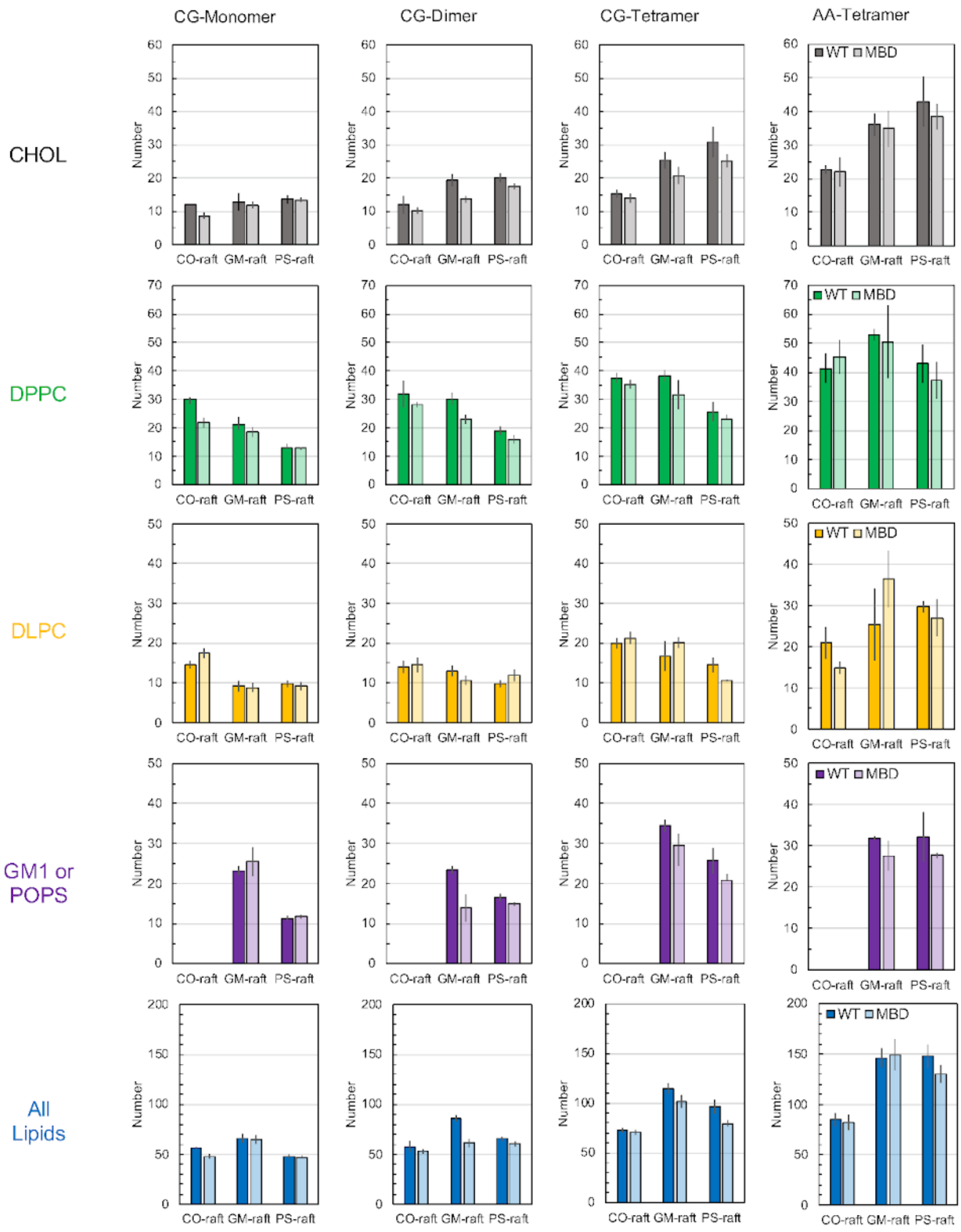

Figure SE1. Number of lipids in the 1.2 nm annular lipid shell. The number of lipids for each lipid type inside the 1.2 nm annular lipid shells surrounding WT-K18 and MBD-K18 oligomers are shown. The number of total lipids for each oligomer at both CG and AA resolutions are also shown. Data points represent time- and replicate averaged values over the last 5 μs for CG or 50 ns for AA and across all three independent replicates of each system. The errors are standard errors of the means.

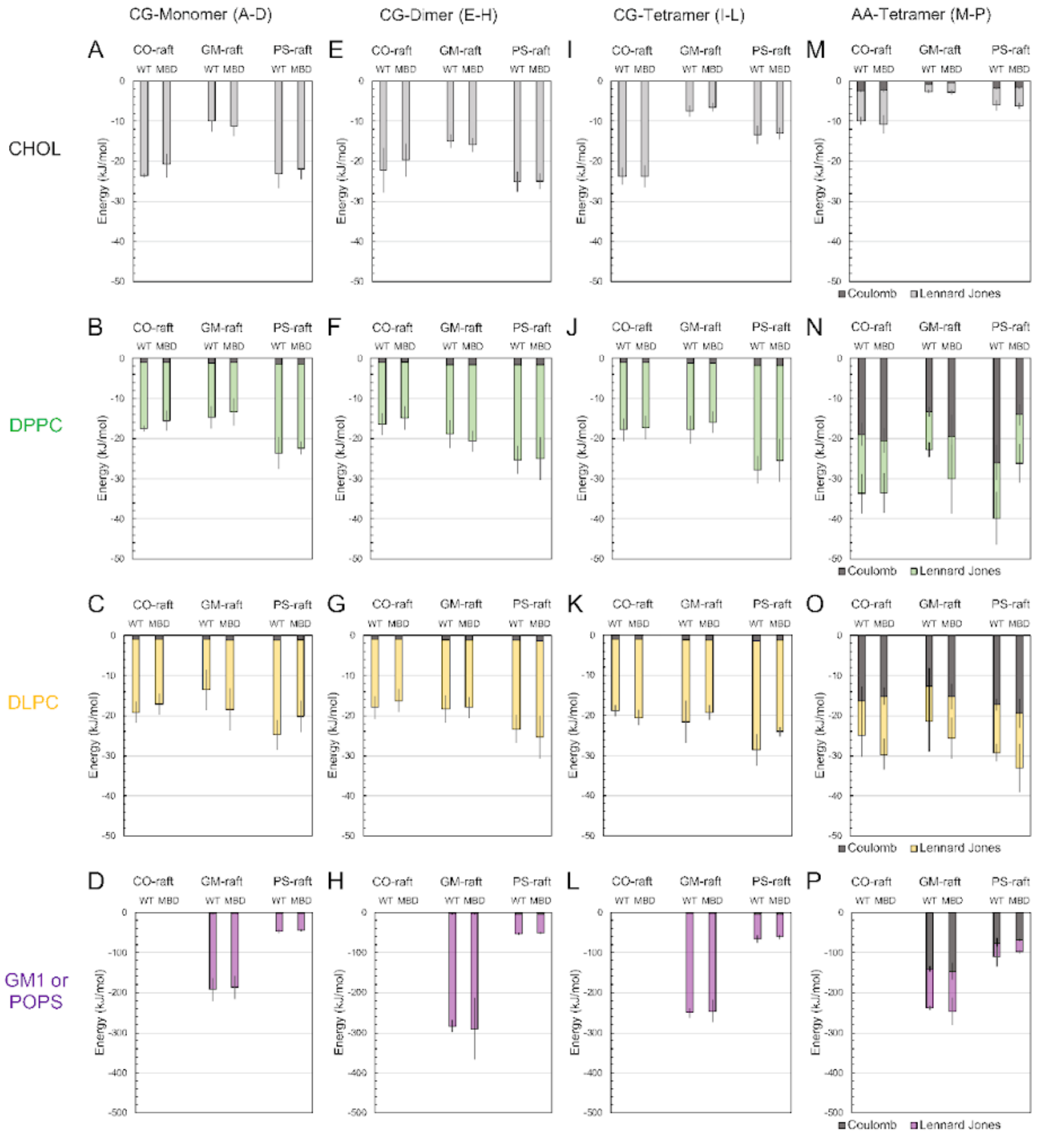

Figure SE2. Normalized protein-lipid binding. The normalized protein-lipid binding of WT-K18 and MBD-K18 oligomers to each lipid type. The data points represent time- and replicate averaged values over the last 5 μs or 50 ns for AA and across all three independent replicates of each system. The error bars are standard errors of the means. See the legend of Fig. 4 for details.

SI - F

DSSP plots of all simulation replicates of AA WT-K18 and MBD-K18 on CO-raft (Fig. F1), GM-raft (Fig. F2) and PS-raft (Fig. F3) are shown. The details of the interpretations of the secondary structures based on DSSP are given in the maintext.

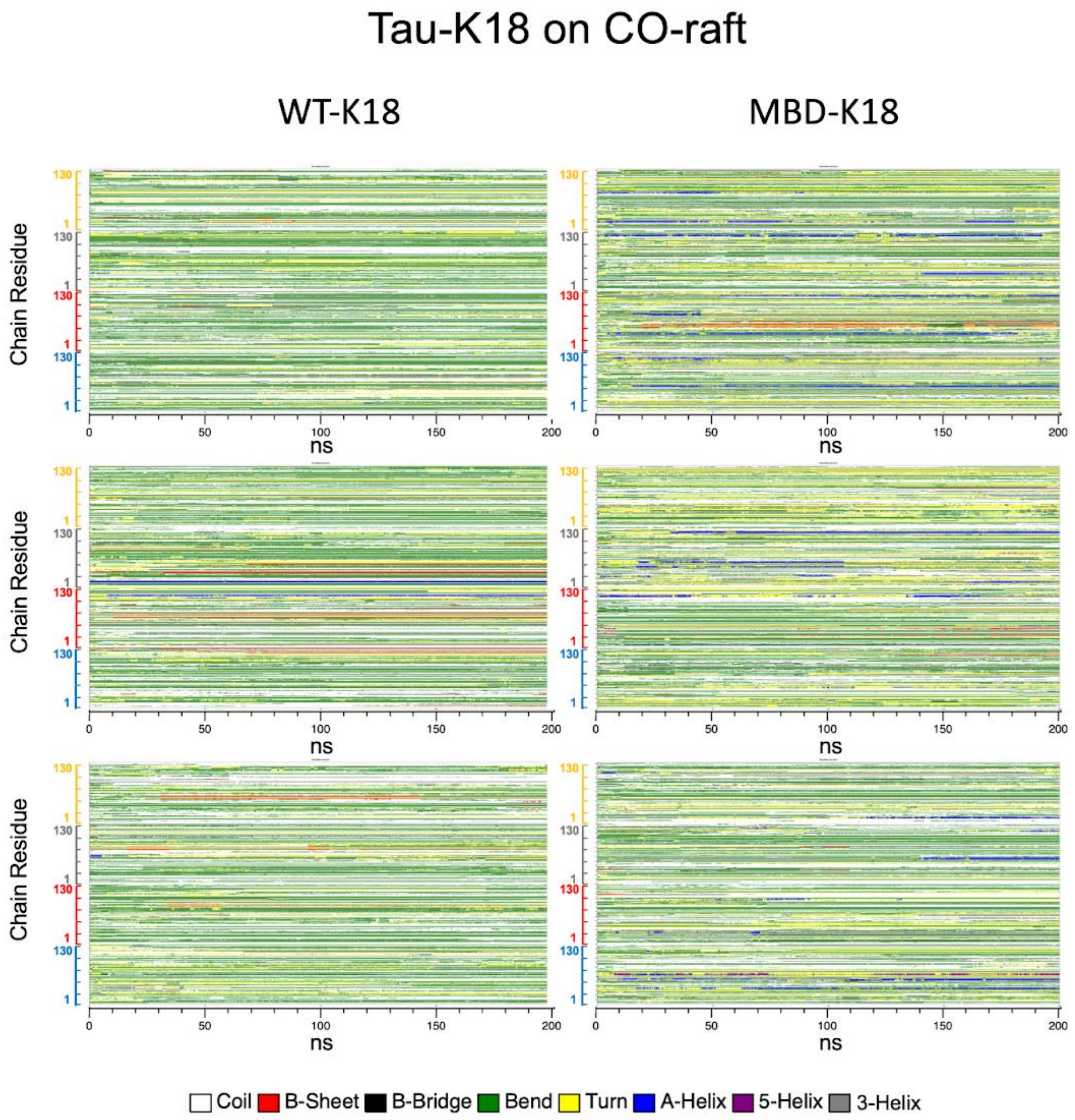

Figure SF1. DSSP plots of WT-K18 and MBD-K18 on CO-raft. See the legend of Fig. 6 for details.

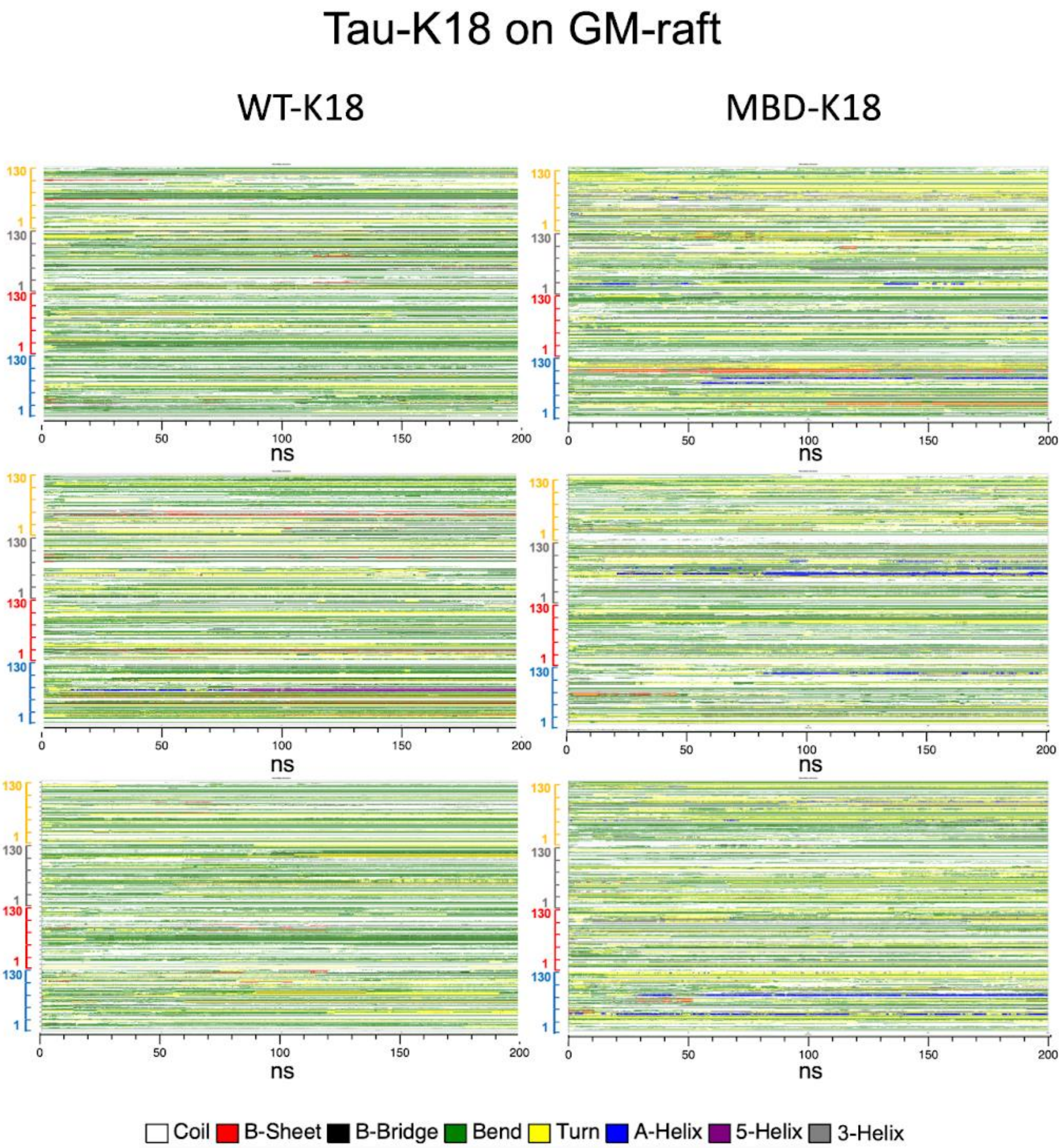

Figure SF2. DSSP plots of WT-K18 and MBD-K18 on GM-raft. See the lengend of Fig. 6 for details.

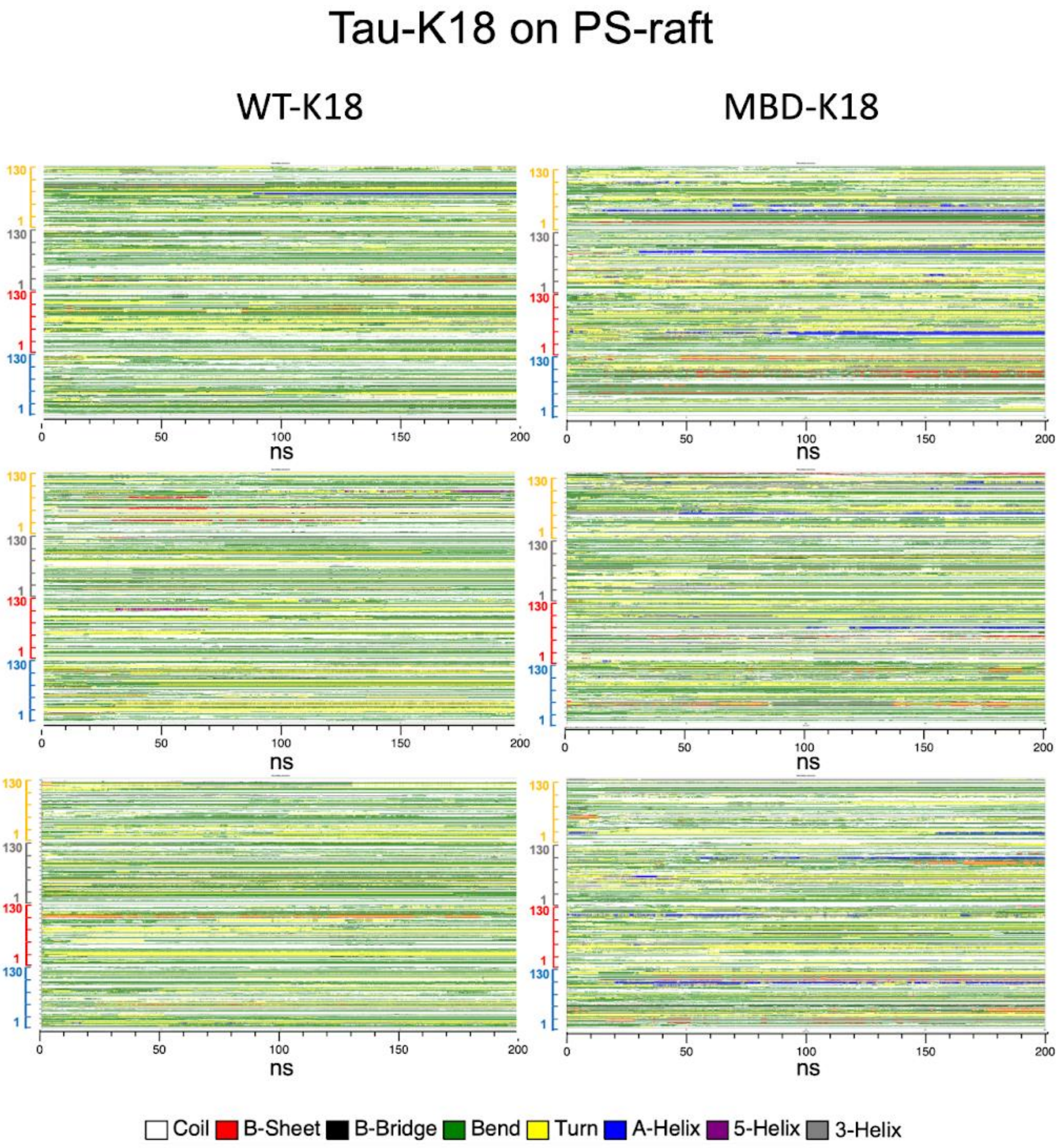

Figure SF3. DSSP of WT-K18 and MBD-K18 on PS-raft. See the legend of Fig. 6 for details.
